# Whole genome assembly of the armored loricariid catfish *Ancistrus triradiatus* highlights herbivory signatures

**DOI:** 10.1007/s00438-022-01947-6

**Published:** 2022-08-25

**Authors:** Alexandre Lemopoulos, Juan I. Montoya-Burgos

**Affiliations:** 1 Department of Genetics and Evolution, University of Geneva, Geneva, Switzerland; 2 iGE3 Institute of Genetics and Genomics of Geneva, Geneva, Switzerland

**Keywords:** Genomics, Loricariidae, Loricarioidei, Herbivory, Freshwater, Vision

## Abstract

The catfish *Ancistrus triradiatus* belongs to the species-rich family Loricariidae. Loricariids display remarkable traits such as herbivory, a benthic lifestyle, the absence of scales but the presence of dermal bony plates. They are exported as ornamental fish worldwide, with escaped fishes becoming a threat locally. Although genetic and phylogenetic studies are continuously increasing and developmental genetic investigations are underway, no genome assembly has been formally proposed for Loricariidae yet. We report a high-quality genome assembly of *Ancistrus triradiatus* using long and short reads, and a newly assembled transcriptome. The genome assembly is composed of 9530 scaffolds, including 85.6% of ray-finned fish BUSCOs, and 26,885 predicted protein-coding genes. The genomic GC content is higher than in other catfishes, reflecting the higher metabolism associated with herbivory. The examination of the SCPP gene family indicates that the genes presumably triggering scale loss when absent, are present in the scaleless *A. triradiatus*, questioning their explanatory role. The analysis of the opsin gene repertoire revealed that gene losses associated to the nocturnal lifestyle of catfishes were not entirely found in *A. triradiatus*, as the UV-sensitive opsin 5 is present. Finally, most gene family expansions were related to immunity except the gamma crystallin gene family which controls pupil shape and sub-aquatic vision. Thus, the genome of *A. triradiatus* reveals that fish herbivory may be related to the photic zone habitat, conditions metabolism, photoreception and visual functions. This genome is the first for the catfish suborder Loricarioidei and will serve as backbone for future genetic, developmental and conservation studies.

**Supplementary Information:**

The online version contains supplementary material available at 10.1007/s00438-022-01947-6.

## Introduction

**Fig. 1:**
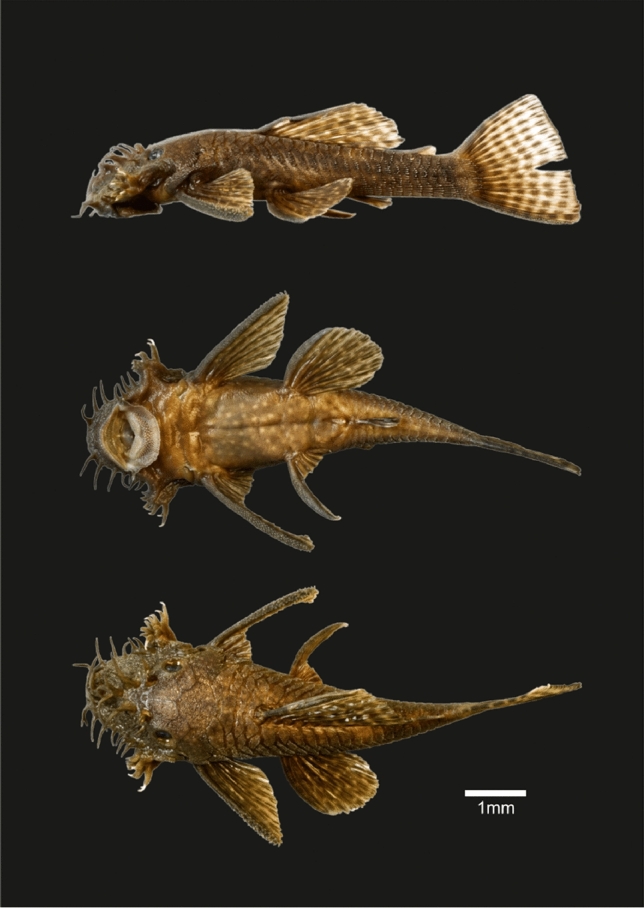
Pictures of the male voucher specimen of *Ancistrus triradiatus* Eigenmann, 1918 used for the whole genome sequencing (voucher number MHNG2786065 belonging to the collection of the Museum of Natural History of Geneva, Switzerland). Lateral (top), ventral (middle) and dorsal (bottom) views. This specimen is 74.21 mm in Standard Length (SL)

The catfish *Ancistrus triradiatus* Eigenmann, 1918 (Fig. 1) belongs to the suckermouth armored catfish family Loricarridae. Loricariids are the largest family of the order Siluriformes, or catfishes (Nelson et al. 2016). This family is endemic to the Neotropics and contains 957 species organized in 120 genera (Froese and Pauly 2011). Loricariids are distinguished from other Siluriformes by a ventral sucker-like mouth, most species are herbivore, and their body is covered with dermal bony plates bearing tooth-like denticles, acting as an exoskeleton (Sire and Huysseune 2003; Rivera-Rivera and Montoya-Burgos 2017).

Loricariids have undergone a subcontinental radiation and have colonized nearly all existing Neotropical freshwater environments (Bail et al. 2012; Covain et al. 2016; Cardoso et al. 2019). The fascinating species diversity characterizing this family, sometimes compared to the African cichlid radiation (Schaefer and Stewart 1993), has yet to be extensively studied, even though some hypotheses about the processes underlying such biodiversity have been proposed (Montoya-Burgos 2003; Jardim de Queiroz et al. 2020; Cardoso et al. 2021). Moreover, only few genomic studies focusing on loricariids have been conducted to date (e.g. Rivera-Rivera and Montoya-Burgos 2018; Roxo et al. 2019).

Loricariids have a substantial economic value, enough to sustain both artisanal and commercial fisheries in South America (Benedito‐Cecilio et al. 2000). Also, due to their worldwide transportation by the ornamental fish trade (Commission for Environmental Cooperation 2009), loricariids have been spread into the wild across the globe and are nowadays considered as invasive species in many countries where considerable resources are invested to control them (e.g. *Pterygoplichthys;* Orfinger et al. 2018).

Within the Loricariidae family, the tribe Ancistrini contains ~ 217 species and 29 genera (Fisch-Muller 2003). With about 65 species described, *Ancistrus* is the species richest genus of Ancistrini (Ferraris 2007; Froese and Pauly 2011). The most prominent characteristics of *Ancistrus* species are the absence of dermal bony plates on the ventral surface and the presence of tentacle-like ornaments on their snout, explaining their common name of bristlenose catfishes. *Ancistrus triradiatus* is a typical representative of the genus, with a body size of ~ 9.2 cm in SL (Froese and Pauly 2011), and inhabiting preferentially clear and shallow waters (the photic zone) of streams and rivers, where they graze on plants, algae and diatoms growing on the substrate. They can be easily kept and bred in captivity.

As most catfishes, *Ancistrus* species have a benthic and mainly nocturnal lifestyle. Yet, *Ancistrus* species are partly active during the day with peak activities at dawn and twilight periods (Buck and Sazima 1995), a behavior that is likely linked to their relatively large and prominent eyes. It has been argued that the benthic and nocturnal lifestyle of catfishes drove the loss of opsin genes involved in daylight vision (Zheng et al. 2021). However, this conclusion was drawn based on the genome of catfish species belonging to the suborder Silurioidei, one out of the two main catfish suborders. Because *Ancistrus* belongs to the second main suborder, the Loricarioidei, the analysis of the opsin genes in *Ancistrus* may serve to verify whether this hypothesis is more broadly valid.

The genus *Ancistrus* is also a good model to assess candidate genes presumably involved in the loss of scales in catfishes, because *Ancistrus* lacks scales, yet most of its trunk is covered with dermal bony plates bearing tooth-like denticles. The SCPP gene family has been shown to play a key role in fish scale formation (Braasch et al. 2016; Liu et al. 2016; Thompson et al. 2021). The loss of specific members of this family (*scpp1* and *scpp5*) was suggested to explain scale loss in catfishes because they were not identified in the genome of the scaleless *Ictalurus punctatus* (Liu et al. 2016). Testing for the presence of genes belonging to the SCPP family in the genome of *Ancistrus* may shed light on the role of this gene family in explaining scale loss along evolution, which has occurred independently in several fish lineages (Lemopoulos and Montoya-Burgos 2021).

The genus *Ancistrus* is also characterized by a large karyotype diversity (Prizon et al. 2016, 2017). Within the genus, chromosome number varies greatly with diploid numbers ranging from 34 to 54 chromosomes (Mariotto et al. 2011; Prizon et al. 2016). It is, however, unclear whether genome duplication may explain such chromosome number variation. Moreover, sex chromosomes are found in some species, with apparently complex sex-determining systems (De Oliveira et al. 2008; Sember et al. 2021), indicating a large structural flexibility in the genomic architecture of this genus.

Here, we report and characterize the first whole genome draft assembly of the herbivorous catfish *Ancistrus triradiatus* based on a combination of high-coverage short Illumina reads, long PacBio reads, and a newly assembled transcriptome. This is the first comprehensive, formal genome assembly of a member of the species-rich family Loricariidae and the suborder Loricarioidei. We describe the genomic characteristics, we examine the composition of two gene families important for scale formation and photoreception and we infer gene family expansions/contractions. We then discuss our findings in relation to the herbivore and photic zone lifestyle of *Ancistrus*. This genome will serve for future studies such as the evolution of the integument, the genetic control of dental tissue formation, the evolution of karyotypes, and for conservation purposes and invasive species control.

## Materials and methods

### De novo transcriptome assembly

#### Samples, library preparation, sequencing

We first assembled de novo the transcriptome of *Ancistrus triradiatus* using a previously generated dataset (Rivera-Rivera et al. 2021). This transcriptome was then used to improve the genome assembly (see hereafter). Quality of single end reads was assessed using FastQC and adaptors were removed using cutadapt (v1.18.; Martin 2011). Trinity v2.11.0 (Grabherr et al. 2013) was used to assemble de novo the transcriptome and the trimmomatic function was used for filtering based on quality and minimum read length (25 bp). The normalization of reads function was not enabled. Finally, putative gene models were inferred through PASA (v2.4.1; Haas et al. 2003) and we used the Trinotate pipeline (v3.2.1; Bryant et al. 2017) for gene prediction and annotation of the transcriptome.

### De novo genome assembly

#### Samples, library preparation, sequencing

Genomic DNA was extracted from a tissue sample of a voucher male specimen of *Ancistrus triradiatus* (Fig. 1)*,* voucher number MHNG2786065 of the Natural History Museum of the City of Geneva collection. Total DNA was extracted using the PeqGold Tissue DNA Mini Kit (PeqLab). The short-read sequencing was done on a HiSeq X Sequencing System (Illumina), and performed at Macrogen Europe (Amsterdam, The Netherlands). The long-read sequencing was done using the PacBio technology (Pacific Biosciences) and performed at the Lausanne Genomic Technologies Facility. The Blue Pippin 15 KB protocol was used, with a 600 min cycles sequencing run, insert size of 30 KB and 1 smrt cell.

#### Genome assembly

Genome size and heterozygosity was estimated based on k-mer count (*k* = 17) using kmerfreq (v.1.0; https://github.com/fanagislab/kmerfreq; Liu et al. 2013a) and the bayes model-based method GCE (genomic character estimator v.1.0.2; https://github.com/fanagislab/GCE; Wang et al. 2020).

To perform the genomic assembly, we first selected the most appropriate assemblers based on three main criteria: (a) they could perform hybrid assemblies using both short and long reads; (b) they could handle medium-sized eukaryotic genomes; (c) they necessitated a moderate amount of computing time and power. Two recent softwares corresponded to our criteria: Wengan (Di Genova et al. 2020) and Haslr (Haghshenas et al. 2020). Due to their recency, limited background was available to evaluate which software suited the best to our sequencing strategy, so we decided to assess both assemblers (Fig. S1).

The Haslr assembly was performed using default parameters. For Wengan v.02, we performed three different assemblies using the different existing algorithms derived from Minia (WenganM; Chikhi and Rizk 2013), Abyss (WenganA; Simpson et al. 2009) and DiscovarDeNovo (WenganD; Weisenfeld et al. 2014). All parameters were set to default except for the -N parameter which was set to -N 2 with regards to the relatively low coverage of our PacBio dataset. The assessment of the assemblies was done based on summary statistics obtained using assemblathon 2 (Bradnam et al. 2013) (Table S1).

To further improve our genome assembly, we used our assembled transcriptome and ran L_RNA_Scaffolder (Xue et al. 2019) with default parameters, which resulted in a refined scaffolding of our genome based on the long RNA contigs (Fig. S1).

Based on the resulting genome assembly, uniqueness of reads was assessed using the dedupe function of the BBmap tools suite (Bushnell 2017). Furthermore, we performed the final cleaning step using kraken2 v2.1.1 to screen for external contamination (Lu and Salzberg 2020). We used the standard database composed of virus, bacteria and human sequences to check for potential contamination in our assembled scaffolds. When contamination was observed, we determined the percentage of contamination within the contaminated scaffolds. We blasted these scaffolds on NCBI using the blastN program (Camacho et al. 2009). If the putative contamination was less than 5% of the total scaffold length, we considered the scaffold as robust and thus did not discard it. If the contamination was ≥ 5%, we discarded the scaffold from our assembly. Finally, our genome assembly completeness was assessed with the Benchmarking Universal Single-Copy Orthologue (BUSCO) approach implemented in BUSCO v.5.2.2 (Seppey et al. 2019). We used the ray-finned fishes (Actinopterygii) *odb10* database, which consists of 26 ray-finned fish species.

#### Genome annotation

Genome annotation was performed using the software *funannotate* (v.1.8.; Palmer 2019). We first produced a masked genome by masking low complexity regions to improve gene prediction and annotation precision. To this end, we produced a de novo repeat library using Repeatmodeler (Flynn et al. 2020) with default parameters. In addition, we used the existing Actinopterygii repeat library to obtain the masked genome through Repeatmasker v.4.1.1 (http://www.repeatmasker.org). Then, we used the masked genome assembly to perform gene predictions using the *funannotate predict* pipeline. To perform gene predictions, we used putative gene models from several sources: our transcriptome, protein evidence (198 reviewed Siluriformes protein sequences and 3500 reviewed zebrafish protein sequences from uniprot-sprot reviewed database). In addition, gene models from *4 *ab initio gene predictors PASA (v2.4. Haas et al. 2003; weight = 2), Augustus/Hi-Q augustus (Keller et al. 2011, weight = 6/8), Snap (Korf 2004; weight = 1) were used for gene model predictions. We finally report the number of protein-coding genes. Functional annotation of the predicted protein-coding genes was performed with default parameters, *i.e.* using funannotate v.1.8 database, which notably includes the databases from Busco (*odb10*; Seppey et al. 2019), Merops (Rawlings et al. 2013) and Pfam (Mistry et al. 2021). Moreover, we used Phobius (default parameters, Käll et al., 2004), eggnog-mapper (v2.0, default parameters; Cantalapiedra et al. 2021), and Interproscan (v. 5.53-87, default parameters; Blum et al. 2021) to assess the biological functions of our predicted protein-coding genes.

#### Genome synteny

To further assess the completeness of our genome assembly and to reveal potential genomic rearrangements in the genome of *Ancistrus triradiatus*, we performed a synteny analysis relative to the genome of a closely related catfish species. We selected the genome of *Ictalurus punctatus* (Liu et al. 2016; GCA_001660625.2) as a reference. We used Last v1.1.33 (Kiełbasa et al. 2011) to perform the synteny analysis and circos v0.69 (Krzywinski et al. 2009) software to visualize the results. This analysis was performed using only the reliably aligned scaffolds longer than 250 KB, corresponding to ~ 50% of the total size of our genome assembly.

#### Gene families

We identified members of the SCPP gene family in the genome of the scaleless *Ancistrus triradiatus* (yet covered with bony plates). The genomic location of the 19 *Ictalurus punctatus* genes of the SCPP family and the *sparcl1* gene were obtained from the information provided in the comparison of these genes between *Ictalurus punctatus* and the bowfin *Amia calva* (Thompson et al. 2021). These *Ictalurus punctatus* gene sequences were then used to identify the corresponding genes in the *Ancistrus triradiatus* genome assembly using tblastX (default parameters, with a threshold e-value of 1E-4 and max_target_seqs 5) (Camacho et al. 2009).

We tested whether the opsin genes that were identified as missing in catfish genomes as a probable consequence of their nocturnal and benthic lifestyle (Zheng et al. 2021) were also lost in *Ancistrus triradiatus*. For this, we searched homologous sequences for a set of 23 visual and non-visual opsin genes usually present in fish genomes. We used the opsin protein sequences from *Silurus meridionalis*, and when absent in this species or ambiguously named, we used the protein sequences of *Danio rerio*. We searched against the *A. triradiatus* genome assembly using tblastn (default parameters, with a threshold e-value of 1e-10 and max_target_seqs 5). As the opsin gene family has many members with similar sequences, most query sequences resulted in good hits with several putative opsin genes in *A. triradiatus*. By crosschecking the best tblasn results over the entire set opsin genes analyzed, we were able to clearly assign the corresponding sequence of *A. triradiatus* to the query sequence of either *Silurus meridionalis* or *Danio rerio.* To confirm the identification of the *A. triradiatus* gene, we took the corresponding tblasn HSPs of each gene, assembled them into a more complete amino acid sequence using the initial query protein sequence as guide, and used blastp to compare it to the NCBI non-redundant protein collection of all actinopterygian species. This procedure provided an independent confirmation of our initial assignation. The list of opsin genes and the GenBank accession number of the protein sequences used as query sequences are presented in the results section.

To further confirm the presence of the *opsin 5* gene in the genome of *A. triradiatus,* a gene that has been considered as absent in other catfish genomes, we verified the synteny of its genomic location in two other closely related species not belonging to the catfish lineage, the zebrafish *Danio rerio* (assembly GRCz11; www.ensemble.org) and the electric eel *Electrophorus electricus* (assembly Ee_SOAP_WITH_SSPACE; www.ensemble.org).

Finally, we investigated gene family expansions and contractions in the genome of *Ancistrus triradiatus*. To perform these analyses, in addition to the predicted protein sequences from our genome, we gathered the predicted protein sequence of the well annotated zebrafish *Danio rerio* (GCA_000002035.4; Howe et al. 2013), the related Characiformes piranha *Pygocentrus nattereri* (GCA_015220715.1; Schartl et al. 2019), and five closely related Siluriformes: the devil’s catfish *Bagarius yarelli* (GCA_005784505.1; Jiang et al. 2019), the Asian red-tail catfish *Hemibagrus wyckiodes* (GCA_019097595.1; Shao et al. 2021), the channel catfish *Ictalurus punctatus* (GCA_001660625.2; Z. Liu et al. 2016)), the Chinese large-mouth catfish *Silurus meridionalis* (GCA_014805685.1; Zheng et al. 2021) and the striped catfish *Pangasionodon hypophtalmus* (GCA_016801045.1; Kim et al. 2018). PorthoMcl (Tabari and Su 2017) was used to predict orthologs and cluster gene families according to sequence similarity (default parameters, 10′000 iterations). To retrace gene family evolution across time, a calibrated tree was extracted from the phylogeny by Rabosky et al. (2018). It was transformed to an ultrametric tree using the *force.ultrametric* function in the phytools package (v1.0.1; Revell 2012) in R environment (v.4.0.3, R Core Team 2020). Then CAFE5 (Mendes et al. 2020) was used to assess expanded and contracted gene families. A global birth and death model was used to assess changes in gene family in each branch and node of the phylogenetic tree (Mendes et al. 2020). The final tree with the gene expansion information was plotted using FigTree (v1.4.3; Rambaut 2016). Gene functional annotation for the ten most significantly expanded gene families (CAFE5 *p* value < 0.05) were assessed using blast2GO (blastp, default parameters; V5.2.5, Conesa et al. 2005).

## Results

### Transcriptome assembly

We assembled de novo the transcriptome of *Ancistrus triradiatus* to improve the genome assembly in a further step. The transcriptome assembly resulted in 180154 transcripts with a N50 length of 1665 bp. (Table 1). The BUSCO analysis revealed that 86.9% of BUSCOs were present and complete in our assembled transcriptome (55.3% single copy, 31.6% duplicated).

**Table 1:** Summary statistics for the final *Ancistrus triradiatus* genome and transcriptome assemblies

	*Ancistrus triradiatus*
Transcriptome assembly	
Number of transcripts	180,154
Total size	153,823,158
N50 length	1665
Largest transcript	65,061
BUSCO scores	
Complete BUSCOs (C)	3161 (86.9%)
Complete and single-copy BUSCOs (S)	3023 (55.3.3%)
Complete and duplicated BUSCOs (D)	1149 (31.6%)
Fragmented BUSCOs (F)	140 (3.8%)
Missing BUSCOs (M)	339 (9.3.%)
Final genome assembly	
Number of scaffolds	9530
Total size of scaffolds	992,310,980
Longest scaffold	3,024,724
Number of scaffolds > 1 K nt	9530
Number of scaffolds > 10 K nt	7974
Number of scaffolds > 100 K nt	2737
Number of scaffolds > 1 M nt	72
Number of scaffolds > 10 M nt	0
Mean scaffold size	104,125
N50 scaffold length	248,302
L50 scaffold count	1012
n90 scaffold length	48,584
L90 scaffold count	4494
scaffold %A	29.62
scaffold %C	20.36
scaffold %G	20.36
scaffold %T	29.63
BUSCO scores	
Complete BUSCOs (C)	3117 (85.6%)
Complete and single-copy BUSCOs (S)	3073 (84.4%)
Complete and duplicated BUSCOs (D)	44 (1.2%)
Fragmented BUSCOs (F)	138 (3.8%)
Missing BUSCOs (M)	385 (10.6%)

Genome was assembled with Wengan v.0.2 (Di Genova et al. 2020), while the transcriptome was assembled using Trinity v2.11 (Grabherr et al. 2013). BUSCO scores were obtained using Busco5 and the odb10 database (Seppey et al. 2019)

### Genome assembly

A total of 50 GB Illumina short sequencing reads were obtained. The long-read PacBio sequencing resulted in 8.2 GB with 640198 P1 reads and a mean read length of 12,812 bp. The initial genome size of *Ancistrus triradiatus* was estimated to be ~ 1.25 GB with the 17-mer depth frequency method and with the bayes model-based GCE method (see “Materials and methods”). Heterozygosity was estimated to be 0.43%. As compared to other catfish genomes, this percentage is comparable to *Silurus meridionalis* (0.49%; Zheng et al. 2021), yet relatively higher than in *Silurus glanis* (0.24%; Ozerov et al. 2020) or *Hemibagrus wyckioides* (0.3%; Shao et al. 2021).

For assembling the genome, we first tested four assembly methods: WenganA, WenganM, WenganD and Haslr (see “Materials and methods”). The first draft assemblies obtained with these four methods were composed of a total number of scaffolds ranging from 12,383 to 42,079 (Table S1). Interestingly, the smallest total size assembly (obtained with Haslr) was also the most fragmented (i.e. highest scaffold number), while the biggest size assembly (obtained with WenganD) was the assembly displaying the fewest scaffolds. Based on the summary statistics (Table S1), the assembly resulting from WenganD was identified as the best one. The information provided by the assembled transcriptome further improved the best selected assembly (WenganD), and reduced the number of scaffolds (from 12,383 to 9641), while also creating longer contigs in general (cf. mean scaffold size, Table S1). The final assembly, obtained after removing the repeated regions and pruning contaminated contigs (following the pipeline presented in Fig. S1 and standard NCBI submission screening), consisted of 9530 scaffolds of a mean size of 104,014 bases. (Table 1).

### Genome annotation and BUSCO analysis

The genomic GC content of *A. triradiatus* is 40.74% (Table 1). Interspersed repeated elements covered ~ 300 MB of the genome, accounting for ~ 33.5% of the genome sequence length (Table S2). This proportion is comparable to other catfish genomes such as *Clarias batrachus* (30.28%; Li et al. 2018) or *Bagarius yarelli* (35.26%; Jiang et al. 2019) but slightly lower than in *Ictalurus punctatus* (41.1%; Liu et al. 2016) or *Clarias magur* (43.72%; Kushwaha et al. 2021). As in other Siluriformes, DNA transposons are particularly abundant in the genome of *A. triradiatus* (accounting for ~ 7% of the genome length), dominated by the Tc1-IS630-Pogo group (Table S2).

The BUSCO analysis revealed that our assembly contains 3,105 (85.6%) complete and 140 (3.8%) fragmented ray-finned fish BUSCOs. Among the complete BUSCOs, 84.4% (3117) are single copy, while 1.2% (44) are duplicated genes. The present BUSCO scores are comparable to the scores reported for the genomes of *Clarias batrachus* (83.9%; Li et al. 2018), *Silurus glanis* (84.2%; Ozerov et al. 2020)*, Pangasianodon hypophtalmus* (89%; Kim et al. 2018) and *Ictalurus punctatus* (89%; Liu et al. 2016), yet somehow lower than values found in the recently assembled genome of *Clarias magur* (95.6%; Kushwaha et al. 2021) or *Silurus meridionalis* (92%; Zheng et al. 2021).

We predicted 26,885 protein-coding genes in our *A. triaradiatus* genome. 91.9% of the genes (24,721) were functionally annotated with putative biological functions. We report a comparable number of predicted protein-coding genes as in other Siluriformes genome annotations such as in *Ictalurus punctatus* (26,661) (Liu et al. 2016) or in *Clarias batrachus* (22,914) (Li et al. 2018).

### Synteny analysis

**Fig. 2:**
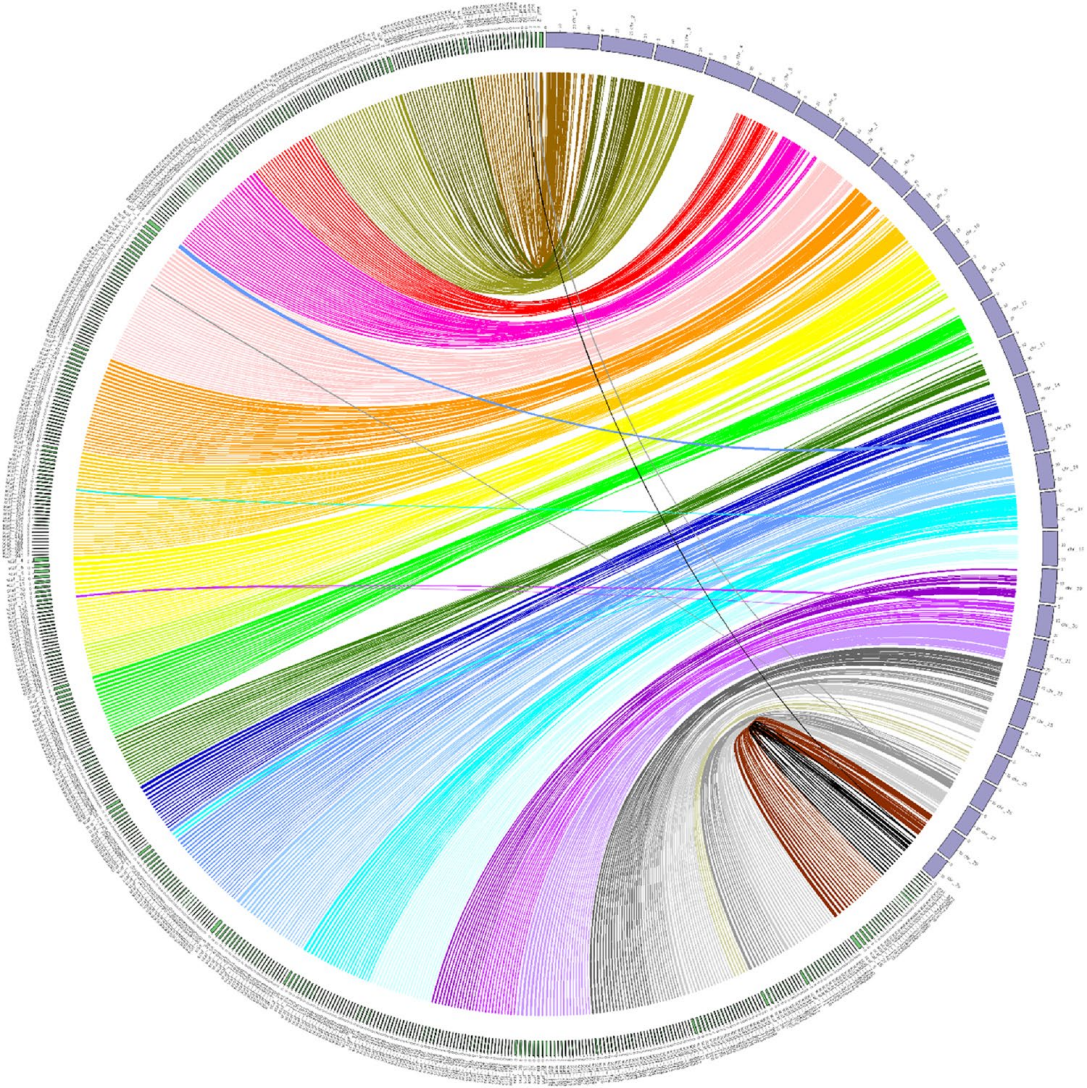
Synteny analysis between the reported genome of *Ancitrus triradiatus* and the genome of *Ictalurus punctatus* (Liu et al. 2016). Only scaffolds > 250 KB are considered for the *A. triraditus* genome assembly (small numbered bars on the left and bottom part of the external circle) while full chromosomes were considered for the *Ictalurus punctatus* genome assembly (numbered grey boxes on the right side of the external circle)

Although our *A. triradiatus* genome comprises more than 9500 scaffolds, some of which being shorter than 250 KB, substantial synteny was found between our assembly and the one of *Ictalurus puncatus* (Liu et al. 2016) (Fig. 2). Notably, all *I. punctatus* chromosomes display abundant regions of homology with *A. triradiatus* scaffolds, including some rearrangements as indicated by the diverging beams in Fig. 2. However, very few synteny links were found between our genome assembly and the chromosome number 4 of *I. punctatus*, which is a sexual chromosome (chromosome X).

### SCPP gene family

We examined the SCPP gene family which is involved in the mineralization process of different tissues, including scales and dermal bones. To this aim, we took the 20 SCPP plus *sparcl1* genes of *Ictalurus punctatus* and used blast to search for best similarity sequences in the genome of *A. triradiatus*. We report the presence of 16 of these genes in our genome assembly; the following 4 genes showed no significant specific match on our assembly: *scpp3a*, *scpp3b5*, *scpp3b6* and *scpp16b* (Table 2). These 16 genes are found in 10 different scaffolds. Interestingly, *scpp3b1*, *scpp3b3* and *odam* cluster in the same scaffold. Both *sparcl1* and *scpp1* are found together in another scaffold. Finally, *spp1* and *scpp8* are located close to each other in another scaffold. These groupings are also observed in the *Ictalurus punctatus* genome assembly (Liu et al. 2016; Thompson et al. 2021) hinting toward a potentially conserved organization of the SCPP gene family in *A. triradiatus*.

**Table 2:**
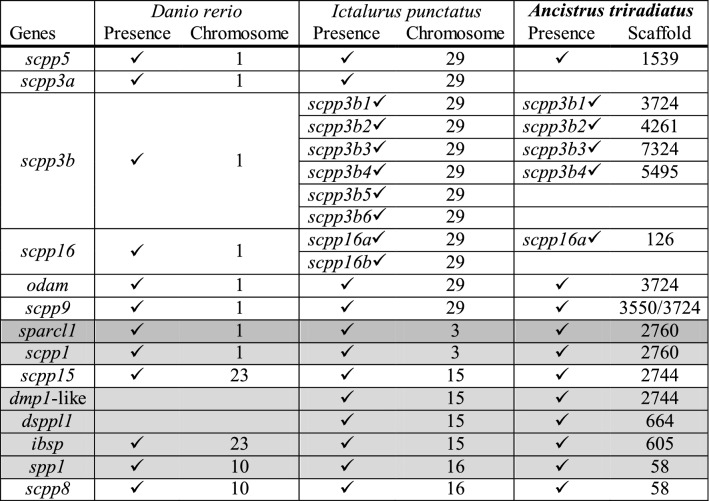
SCPP gene family in *Ancistrus triradiatus* as compared to two reference species, *Danio rerio* and *Ictalurus punctatus*

The acidic *scpp* genes are represented in white, while P/Q rich genes are illustrated in light grey. The gene *sparcl* is represented in dark grey. Data for *Danio rerio* and *Ictalurus punctatus* comes from Thompson et al. (2021). *Ancistrus triradiatus* scaffolds correspond to the assembled genome reported in this study. The *scpp3b* gene has undergone gene duplications in both catfish species while two copies of the *scpp16* are found in *Ictalurus punctatus.* For *scpp9*, blast searches yielded two significant hits with similar scores against the *Ancistrus triradiatus* assembly (in scaffolds 3550 and 3724), suggesting a gene duplication in this species (we note that the position of the *scpp9* blast hit on scaffold 3724 is different from the position of the blast hit of *scpp3b1* on this same scaffold)

### Opsin gene family

We examined whether the genes of the opsin family that have been identified as absent in other catfish genomes were also absent in our *A. triradiatus* genome assembly. For this aim, we tested a panel of 23 visual and non-visual opsins genes usually present in fish genomes by comparing the protein sequences of *Silurus meridionalis*, and when absent in this species, the protein sequences of *Danio rerio*, against the genome of *A. triradiatus*. The best tblastn results providing an initial assignation together with the confirmation results obtained by comparing the *A. triradiatus* HSPs against the NCBI non-redundant protein collection of actinopterygians are given in Table S3.

From the set of 23 *opsin* genes, we detected the presence of 18 of them in *A. triradiatus* and report their specific location in our genome assembly (Table 3). Six opsin genes were not found in the genome of *A. triradiatus* (*sws1*, *sws2*, *tmtops2a*, *tmtops3a, tmtops3b,* and *opsin 9*), all six being also absent from the genome of the reference catfish *S. meridionalis* (Table 3). The nomenclature of the *tmtops* paralogous genes being confusing, we clarified it by inferring the *tmtops* phylogeny including all the known copies of *Danio rerio*, the copies of four well annotated catfish genomes plus the copies of *A. triradiatus* and of two related Teleostei (Fig. S3). We were thus able to see that all the catfish species tested share the presence of three *tmtops* genes in their genome: *tmtopsa*, *tmtoposb*, and *tmtops2b* (Fig. S3 and Table S4).

**Table 3:**
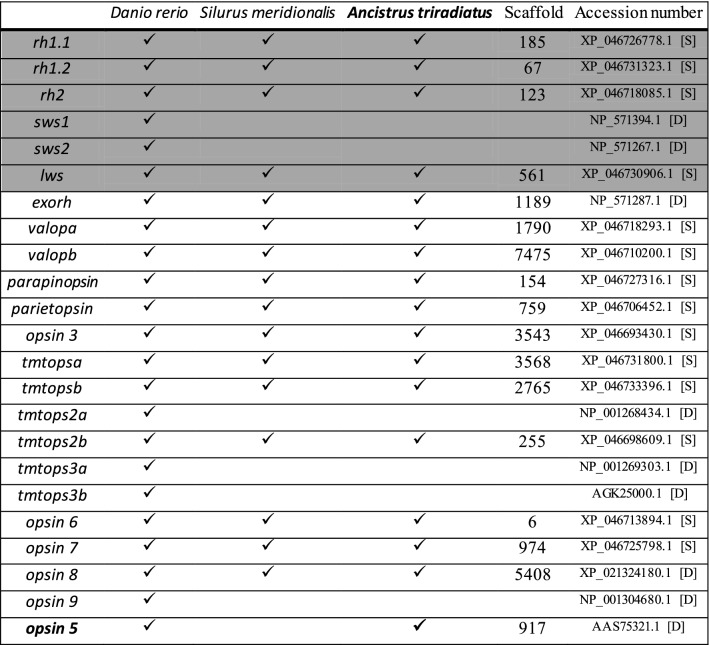
Opsin gene repertoire in *Ancistrus triradiatus* as compared to two reference species, *Danio rerio* and *Silurus meridionalis*

The protein sequences of *S. meridionalis*, and when absent, of *D. rerio*, where used as query sequences for the detection of homologs in *A. triradiatus*. The query sequence accession number is given in the last column, with [S] indicating *Silurus* and [D] indicating *Danio*. The visual opsin genes are represented in grey, while the non-visual opsins are represented in white. The scaffold numbering corresponds to the *A. triradiatus* genome assembled in this study. Interestingly, *A. triradiatus* has not lost *opsin 5* (in bold), in contrast to other catfishes such as *Silurus meridionalis.* Opsin genes classification and nomenclature are based on Zheng et al. (2021), excepting the *tmtops* genes which classification was revised based on our phylogenetic analysis (Fig. S3)

Interestingly, we detected the presence of *opsin 5* in the genome of *A. triradiatus* (the amino acid sequence is given in Table S4), while this gene is absent from the genome of the catfish *S. meridionalis*. To validate the presence of *opsin 5* in the genome of *A. triradiatus*, we tested its synteny relative to other closely related non-catfish species by verifying the presence of the neighboring genes in the genome of *Danio rerio* and the electric eel *Electrophorus electricus.* In the genome of these two reference species as in the genome of *A. triradiatus*, the *opsin 5* gene is flanked by the genes *ptchd4* and *cd2dap,* thus confirming the presence of *opsin 5* in *A. triradiatus.*

### Gene family expansion and contractions

**Fig. 3:**
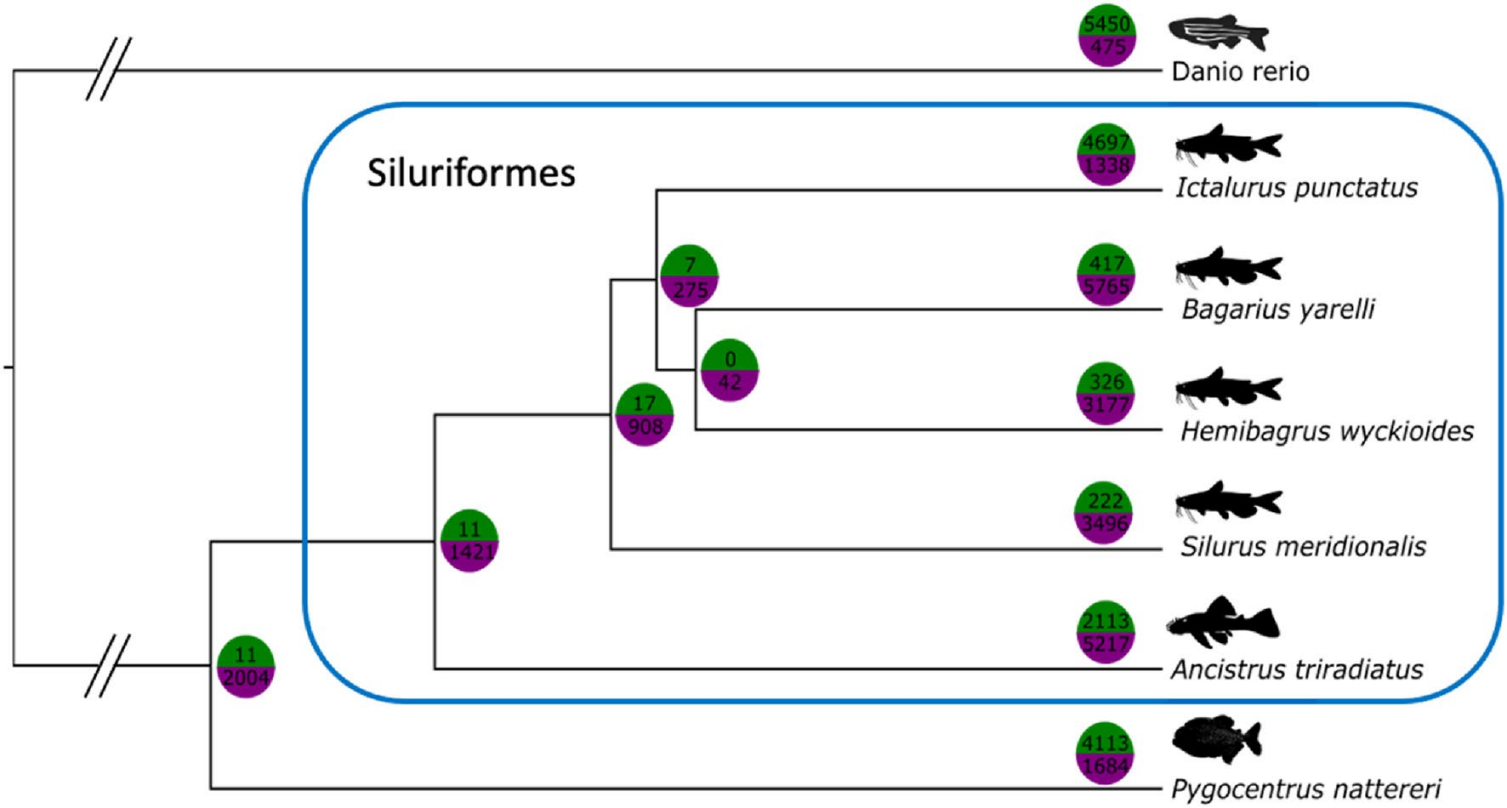
Gene family expansion and contraction in seven actinopterygian species related to *Ancistrus triradiatus*. The number of expanded gene families is shown in green (upper part of the circle), while the number of contracted gene families is shown in purple (lower part of the circle). Gene family expansion/contraction analysis was performed with CAFE5 (Mendes et al. 2020), and using previously published genomes (see “Matherials and methods”). The phylogenetic tree was extracted from the actinopterygian phylogeny of Rabosky et al. (2018)

Our analysis shows that within the Siluriformes, there are generally much more gene family contractions than expansions, except for *Ictalurus punctatus* (Fig. 3). Moreover, most gene family expansions and contractions were inferred as lineage-specific events, i.e. along the terminal branches of the tree. Interestingly, *A. triradiatus* shows the largest amount of gene family changes (expansions plus contractions). In this species, 2123 gene families were expanded and 5217 were contracted (Fig. 3), with 163 families (53 expanded/110 contracted) being statistically significant*.* The functional annotations of the ten most significantly expanded families indicate that they are mainly involved in immune system processes (Table S5). The single exception is the gamma crystallin gene family which is involved in eye lens formation.

## Discussion

We report and examine the genome of the catfish *Ancistrus triradiatus*, belonging to the species-rich family Loricariidae and the suborder Loricarioidei. Although the Loricarioidei suborder comprises ~ 41% of all catfish species of the world (Moreira et al. 2017), this is the first formal whole genome assembly of a Loricarioidei representative, thus reducing a knowledge gap.

### Genome characteristics

The genome size of *A. triradiatus* was estimated to be ~ 1.25 GB, while the size of the assembled genome is 992 MB. This size is comparable, yet in the higher end, to the genome size of the eight other Siluriformes species reported to date: ∼600 MB for *Bagarius yarelli* (Jiang et al. 2019), ~ 700 MB for *Pangasionodon hypophtalmus* (Kim et al. 2018), ~ 741 MB for *Silurus meridionalis* (Zheng et al. 2021), ∼779 MB for *Hemibagrus wyckiodes* (Shao et al. 2021), ~ 800 MB for *Silurus glanis* (Ozerov et al. 2020), ~ 900 MB for *Clarias batrachus* (Li et al. 2018), ~ 1 GB for *Clarias magur* (Kushwaha et al. 2021) and ~ 1 GB for *Ictalurus punctatus* (Liu et al. 2016). This result gives no clear support for a whole genome duplication event along the evolution of the *Ancistrus* lineage, despite the marked chromosome number variation found in this genus and in the family to which it belongs, the Loricariidae (Prizon et al. 2016; Deon et al. 2022). Moreover, considering that a recent whole genome duplication would imply finding most genes in two copies, the small proportion of duplicated BUSCOs in the genome of *A. triradiatus* (only 1.2% of all BUSCOs are duplicated) provides an additional support for the absence of a recent lineage-specific whole genome duplication.

Among the reported catfish genomes, the slightly larger genome sizes of the species *A. triradiatus*, *I. punctatus* and *C. magur* tend to correlate with more predicted protein-coding genes, with the notable exception of *P. hypophtalmus* which has the largest number of protein-coding genes (28,580) while its genome is ~ 700 MB (Kim et al. 2018). Nevertheless, there is a significant positive linear regression between the estimated genome size and the predicted number of protein-coding genes based on the eight published whole genomes of Siluriformes plus the genome of *A. triradiatus* we report (*R*^2^ = 0.97, *F*(1,8) = 232.6, *P* < 0.001; setting *Y*-intercept at 0) (Fig. S2). A positive relationship between genome size and gene content has been documented for a collection of species, and this relationship indicates that gene content tends to increase slower as genome size increases (Hou and Lin 2009).

Our synteny analysis between the genomes of *A. triradiatus* and *Ictalurus punctatus* revealed some rearrangement events, which are not surprising given the ~ 140 Ma long evolutionary history separating the families to which these two species belong: the Loricariidae and the Ictaluridae, respectively (Rivera-Rivera and Montoya-Burgos 2017). In addition, chromosome number variation and evidence of chromosome rearrangements have been reported in the genus *Ancistrus* (Mariotto et al. 2011; Prizon et al. 2016). The synteny analysis also showed a virtual absence of links between our *A. triradiatus* genome assembly and the sexual chromosome X (chromosome 4) of *Ictalurus punctatus* (Fig. 2). One could ask whether this observation may be explained by the sex of the sequenced individuals, the *Ictalurus punctatus* genome coming from a female individual, while the *A. triradiatus* specimen we sequenced was a male. *I. punctatus* has a sex-determination system in which females are homogametic (females XX and males XY) (Bao et al. 2019). However*,* the absence of the Y chromosome in the genome of *I. punctatus* cannot account for this lack of homology as the recently sequenced Y chromosome of *I. punctatus* has essentially no sequence difference with the X chromosome (Bao et al. 2019). The most probable explanation lies in the difference between sexual chromosome systems in these two species. In *Ancistrus,* sexual chromosome systems are still poorly understood but seem very complex, with the existence of systems ranging from no sex chromosome to multiple sex chromosomes (XX/XY1Y2 and Z1Z1Z2Z2/Z1Z2W1W2) (De Oliveira et al. 2008). Further investigations are needed to decipher the sex chromosome system in *A. triradiatus*, which is still unknown. Our synteny analysis is, however, incomplete, because only ~ 50% of the total size of our genome assembly was used (only scaffolds > 250 KB were included in the analysis).

### Higher GC content in the herbivorous *A. triradiatus*

The genome of *A. triradiatus* has a higher GC content (40.74%) than in other Siluriformes genomes: 39.83% in *Clarias magur* (Kushwaha et al. 2021), 39.2% in *Clarias batrachus* (Li et al. 2018), 39% in *Ictalurus punctatus* (Liu et al. 2016), 38.3% in *Pangasianodon hypophtalamus* (Kim et al. 2018), or 39% in *Silurus meridionalis* (Zheng et al. 2021). Other non-Siluriformes fish groups may have lower genomic GC content such as in the Cypriniformes *Cyprinus carpio* (37.0%) and *Danio rerio* (36.6%), or markedly higher like in the Gasterosteiformes *Gasterosteus aculeatus* (44.6%) and the Tetraodontiformes *Takifugu rubripes* (45.5%) and *Tetraodon nigroviridis* (46.4%) (Xu et al. 2014). The heterogeneous genomic GC content in fish has been previously observed and explained in part by the role of environment factors and lifestyles, with freshwater and sedentary species displaying the lowest GC content, providing support to the metabolic rate hypothesis as the main driver of genomic GC content (Tarallo et al. 2016). According to this general rule, the slightly higher GC content found in the genome of *A. triradiatus* relative to other catfishes coincides with the characteristics of this small and active species inhabiting rheophilic freshwater habitats, indicative of a higher metabolic rate. Moreover, it has been shown that in vertebrates, herbivory leads to higher basal metabolic rates (Clarke and O’Connor 2014). As all other catfish species compared are omnivores or carnivores, the plant-based diet of *Ancistrus* provides an additional support for the metabolic rate explanation of the higher GC content in the genome of *A. triradiatus*.

### Scale loss is not driven by specific SCPP gene losses

The SCPP gene family has been extensively discussed in relation to its important role in the mineralization of different tissues, including scales and dermal bones. SCPP genes are divided into two subclasses: the acidic genes, which play a role in bone and dentin mineralization, and the P/Q rich genes, associated with enamel formation (for more details, see Kawasaki 2011; Lv et al. 2017). SCPP genes have thus been suggested to be critical for scale formation and scale diversity in fish (Braasch et al. 2016; Liu et al. 2016; Thompson et al. 2021). Interestingly, Liu et al. (2016) proposed two candidate genes for scale formation, *scpp1* and *scpp5*, as they were unidentified in the genome of the scaleless catfish *Ictalurus punctatus* in contrast with other scaled fish species. Moreover, *scpp1* and *scpp5* were present in the preliminary draft genome assembly of two “scaled catfish” species, *Platydoras armatulus* and *Pterygoplichthys pardalis* (Liu et al. 2016). However, these two catfish species are in fact scaleless, as their integument is devoid of scales, yet partly of fully covered with dermal bony plates. Dermal bony plates are mineralized structures found in several fish species (Sire and Huysseune 2003; Vickaryous and Sire 2009) and never co-occur in combination with scales (Lemopoulos and Montoya-Burgos 2021). Moreover, a recent study showed that *scpp1* and *scpp5* genes were actually present in the scaleless *Ictalurus punctatus* (Thompson et al. 2021). Our results indicate that *scpp1* and *scpp5* genes are also present in the genome of the scaleless *A. triradiatus*. Taken together, these findings indicating that *scpp1* and *scpp5* cannot be considered as candidate genes driving scale loss when they are absent and scale formation when they are present in the genome.

### Opsin gene repertoire in a photic zone catfish

The benthic and nocturnal lifestyle of catfishes has been proposed to be the cause of gene losses in the opsin gene family, which are otherwise present in diurnal fishes inhabiting clear and shallow waters (Zheng et al. 2021). In the analysis of Zheng et al. (2021), the catfish representatives all belong to the suborder Siluroidei. To test whether this hypothesis holds true for non-Siluroidei catfishes, we examined a set of 23 opsin genes in the genome of *A. triradiatus*. Our findings indicate that six of them are absent in the genome of *A. triradiatus* (*sws1*, *sws2*, *tmtops2a*, *tmtops3a, tmtops3b,* and *opsin 9*), all of which are also absent in the genome of the Siluroidei species analyzed by Zheng et al. (2021). Consequently, these gene losses most probably occurred before the divergence of the two catfish suborders Siluroidei and Loricarioidei. Because two of the lost genes are visual opsins sensitive to ultraviolet and blue violet light (*sws1* and *sws2*), and because ultraviolet wavelengths cannot penetrate deep into the water column and blue violet wavelengths are more easily scattered by suspended particles in turbid waters, Zheng et al. (2021) concluded that the loss of these two genes may be an adaptation to bottom and turbid water environments. The same argument was proposed by Zheng et al. (2021) to explain the loss of two other UV-sensitive yet non-visual opsin genes in Siluroidei catfishes, *opsin 5* and *tmt2.*

In the genome of *A. triradiatus*, we identified the presence of the *opsin 5* gene, which we confirmed by a synteny analysis including well annotated genomes of related species. *Opsin 5* encodes a UV-sensitive photoreceptive protein (Yamashita et al. 2010, 2014; Kojima et al. 2011) present in in most vertebrate lineages (Hankins et al. 2014). In mammals, *opsin 5* it is the most conserved gene of the opsin family (Upton et al. 2021). The Opsin 5 protein is a key player in functions such as local clock entrainment in mouse (Buhr et al. 2019), photoperiod detection in birds (Nakane et al. 2014) or light avoidance in *Xenopus* tadpoles (Currie et al. 2016). Several paralogous *opsin 5*-like copies have been found in zebrafish (Davies et al. 2015), leading to the discovery of orthologs in many vertebrates, which have been organized into six groups: *opsin 5*, *opsin 6*, *opsin 7a*, *opsin 7b*, *opsin 8* and *opsin 9* (Beaudry et al. 2017). Functional analyses have been performed on the opsin proteins encoded by some of these genes, including Opsin 8 (= Opn5L2) (Ohuchi et al. 2012), Opsin 9 (= Opn5m2) (Sato et al. 2016), and Opsin 7a (= Opn5L1a) (Sato et al. 2018; Sakai et al. 2022), indicating that they are also sensitive to UV-light.

Although the Siluroidei catfishes analyzed by Zheng et al. (2021) do not possess the *opsin 5* gene, they do have other UV-sensitive *opsin 5*-like genes such as *opsin 7* and *opsin 8.* This observation suggests that the benthic and nocturnal Siluroidei catfishes still need to sense UV-light for functional reasons other than vision, because their UV-sensitive non-visual opsin gene repertoire has not been entirely lost. The fact that *opsin 5* is present in the genome of *A. triradiatus* suggests first that the loss of this gene in the Siluroidei catfish species analyzed by Zheng et al. (2021) occurred after the divergence of the Siluroidei and Loricarioidei catfish suborders. Second, the presence of *opsin 5* in the genome of *A. triradiatus* indicates that sensing UV light has a greater functional relevance in this species than in the nocturnal catfishes lacking this gene. This is congruent with the ecology and lifestyle of *Ancistrus,* whose species are herbivorous and inhabit the photic zone of rivers where sub-aquatic vegetation grows. They also display complex nictemeral and seasonal behaviors, as well as size-dependent depth preferences and hiding-in-the-dark strategies related to nocturnal and diurnal predator avoidance (Buck and Sazima 1995; Power 2003), activities that benefit from a precise light sensing capacity.

### Immune gene family expansions

The large amount of gene family changes (expansions plus contractions) we found in *A. triradiatus* correlates with the high chromosome number variation and rearrangements characterizing this genus (Mariotto et al. 2011; Prizon et al. 2016). Genomic reorganizations may occasionally replicate or delete genes generating new gene copy number variants, offering new opportunities to improve cellular or physiological functions. Because new maladapted gene copy number variants tend to disappear via purifying selection and variants with no fitness effect undergo genetic drift without any trend for increasing (or decreasing) gene copy number, significantly expanded (or contracted) gene families are thus interpreted as resulting from the process of adaptive evolution acting on gene copy number. Our analysis of the ten most significantly expanded families in the genome of *A. triradiatus,* as compared to related fish species, shows that they are involved in immune functions. This finding is consistent with the fact that genes of the immune system are recognized to be common targets of natural selection, as they are involved in the host–pathogen arms race (Shultz and Sackton 2019). However, why a particular species like *A. triradiatus* would undergo significant lineage-specific remodeling of immune gene families? There could be several explanations to this result. First, we could hypothesize that an improved immune response would be necessary in typical scaleless, benthic species such as *Ancistrus,* inhabiting microbial-rich habitats (Holm et al. 2017; Lemopoulos and Montoya-Burgos 2021). We could also speculate that the typical warm waters where *Ancistrus* is found are conditions favoring pathogen proliferation, magnifying the challenge on the immune system (Sun et al. 2020). However, the other catfish species compared share the same scaleless integument and benthic ecology with *A. triradiatus*. Moreover, all the species compared in our analysis live in relatively warm waters, apparently precluding these explanations. Nevertheless, adaptive evolution in host–pathogen interaction systems goes fast and occurs independently across lineages, leading to frequent lineage-specific expansions of different immune gene families, as documented in vertebrates (Lutfalla et al. 2003; Liu et al. 2013b).

### Gamma crystallin family expansion for a better vision

The gamma crystallin gene family, which is involved in eye lens formation, was significantly expanded in *A. triradiatus*. This gene family contains more members in fishes as compared to other non-aquatic vertebrates (Mahler et al. 2013; Chen et al. 2016; Wistow and Slingsby 2016), most probably because sub-aquatic vision requires a specific lens structure (harder and more dense) due to the high retraction index in this environment (Wistow and Slingsby 2016). The expansion of the gamma crystallin gene family in fishes may have thus played a crucial role in improving lens composition for a better sub-aquatic vision (Wistow and Slingsby 2016). Moreover, lens characteristics and composition directly impact the pupil shape (Land 2006). Loricariids possess a remarkable crescent-shaped pupil which has independently evolved in other vertebrates such as sharks and rays, some bottom-dwelling actinopterygians, and some terrestrial species (Murphy and Howland 1991). Such pupil shapes have functional effects like a larger visual field or an enhanced contrast at high spatial frequencies (Murphy and Howland 1991; Douglas et al. 2002). In addition, it has been shown that elaborated crescent pupils may have a camouflage role in bottom-dwelling skates (Youn et al. 2019), and a similar role has been hypothesized for the bottom-dwelling loricariids (Douglas et al. 2002). As such, it is probable that the expansion of the gamma crystallin gene family we discovered in *A. triradiatus* is associated with its crescent-shaped pupil. The larger gamma crystallin gene family and the sophisticated pupil found in *A. triradiatus* are most probably linked to its herbivorous diet which constrains it to live in the photic zone of rivers where vegetation can grow. Although the feeding activity of *Ancistrus* is higher at dawn and twilight periods, foraging also occurs during the day (Buck and Sazima 1995). This diurnal activity in the photic zone matches with the sophisticated pupil and larger eye size in *Ancistrus*, and contrasts with the standard pupils and small eyes found in the nocturnal catfishes of the suborder Siluroidei (Zheng et al. 2021). The expansion of the gamma crystallin gene family we revealed is thus likely related to a better sub-aquatic vision in *A. triradiatus* driven by its herbivore diet, as compared to other catfishes.

## Conclusion

We sequenced, assembled and characterized the genome of the catfish *Ancistrus triradiatus*, which is the first formal whole genome assembly of a representative of the species-rich Loricariidae family and Loricarioidei suborder. Despite the large karyotype variability characterizing this genus, we found no evidence supporting a recent lineage-specific whole genome duplication. Our synteny analysis between *A. triradiatus* and *I. punctatus* indicates major differences in sexual chromosome content, highlighting complex and understudied sexual chromosome systems. The genome of *A. triradiatus* displays the highest GC content relative to the genome of other catfish species, which is a genomic signature of higher metabolic rates (according to the metabolic rate hypothesis) associated with herbivory. We scrutinized the SCPP gene family to test the explanatory role of specific gene losses as drivers of scale loss in fish but found the presence of the candidate genes in the genome of the scaleless *A. triradiatus,* refuting the SCPP gene loss hypothesis. Our new genome assembly allowed the examination of the gene repertoire of the photoreceptive opsin proteins in a non-Siluroidei catfish species, a repertoire that has been reduced in Siluroidei species presumably due to their benthic and nocturnal lifestyle. Our result indicates that the well conserved *opsin 5* gene encoding a UV-light-sensitive opsin was not lost in *A. triradiatus*, contrasting with its loss in Siluroidei representatives. This finding indicates that sensing UV-light variations is functionally more relevant in the herbivorous *A. triradiatus*, which inhabits the photic zone of rivers, than in benthic and nocturnal Siluroidei catfishes inhabiting turbid waters. Another genomic feature which may be related to the herbivorous lifestyle of *A. triradiatus* is the expansion of the gamma crystallin gene family, which contributes to the structure of the lens and conditions the pupil’s shape, modulating sub-aquatic vision. Thus, our analysis of the genome of the catfish *A. triradiatus* reveals that herbivory, which is related to the photic zone habitat, would condition metabolism, photoreception and visual functions in fish. We note that herbivory is a complex behavior controlled by many genetic factors, and the genomic features we highlighted here are likely consequences of this behavior. This genome is a new resource for future evolutionary studies, such as the evolution of the integument, the genetic regulation of dental tissue emergence on the trunk or variations in chromosome number and structure. It will also be useful in research focusing on fish conservation, invasive species control, and in aquaculture genomics.

## Supplementary Information

Below is the link to the electronic supplementary material.

Supplementary file1 (DOCX 395 KB)

## Data Availability

The raw RNA data are accessible at NCBI SRA repository under the accession number PRJNA733776. The assembled genome and its annotation are available on NCBI, under accession number JAJAGP000000000. The version described in this paper is version JAJAGP010000000.
