# Supplementary material for: Whole genome assembly of the armored loricariid catfish Ancistrus triradiatus highlights herbivory signatures
Source: Mol Genet Genomics. 2022 Aug 25;297(6):1627–42. doi: 10.1007/s00438-022-01947-6 (PMC9596584; doi:10.1007/s00438-022-01947-6)
Supplement: Supplementary file 1 — Supplementary file1 (DOCX 395 KB) [file 438_2022_1947_MOESM1_ESM.docx]

SUPPLEMENTARY INFORMATION

Title: **Whole genome assembly of the armored loricariid catfish *Ancistrus triradiatus* highlights herbivory signatures**

Journal: **Molecular Genetics and Genomics**

Authors: Alexandre Lemopoulos^1^, Juan I. Montoya-Burgos^1,2,3^

1. Department of Genetics and Evolution. University of Geneva, Geneva, Switzerland
2. iGE3 institute of Genetics and Genomics of Geneva
3. E-mail: [Juan.Montoya@unige.ch](mailto:Juan.Montoya@unige.ch)

**List of Tables and Figures**

**Table S1**. Summary statistics for four different *de novo* genome assemblies.

**Table S2**. Repeated elements in the *Ancistrus triradiatus* genome assembly.

**Table S3.** Determining the presence or absence of members of the opsin gene family in the genome of *Ancistrus triradiatus*.

**Table S4**. Predicted amino acid sequences of the four genes belonging to the opsin family in *Ancistrus triradiatus* that we analyzed more specifically: *tmtopsa*, *tmtopsb*, *tmtops2b*, and *opsin 5.*

**Table S5**. Description of the members of the 10 most significantly expanded gene families in *Ancistrus triradiatus*.

**Fig. S1**. Flowchart depicting the steps performed before obtaining the final genome assembly.

**Fig. S2**. Positive linear regression between the estimated genome size and the predicted number of protein-coding genes in Siluriformes.

**Fig. S3**. Maximum-likelihood tree of the protein sequences of the *teleost multiple tissue opsin* (*tmtops*) genes from catfish species and selected relatives.

**Table S1**. Summary statistics for four different *de novo* genome assemblies. WenganM, WeganA and WenganD were obtained through the three different modes of the Wengan software (Di Genova et al. 2020), i.e. Minia3, Abyss2 and DiscovarDenovo. Haslr assembly was obtained using the haslr software (Haghshenas et al. 2020). The LRNA column assembly was obtained using the WenganD assembly combined with long RNA reads, using L_RNA_Scaffolder (Xue et al. 2019).

|  | WenganM | WenganA | WenganD | Haslr | LRNA (WenganD) |
| --- | --- | --- | --- | --- | --- |
| Number of scaffolds | 15741 | 14788 | 12383 | 42079 | 9641 |
| Total size of scaffolds | 847546682 | 857055366 | 995311060 | 699035291 | 995585260 |
| Longest scaffold | 1585797 | 1608687 | 2054530 | 237890 | 3024724 |
| Number of scaffolds > 1K nt | 15741 | 14788 | 12383 | 42027 | 9641 |
| Number of scaffolds > 10K nt | 12871 | 12202 | 10499 | 21794 | 8056 |
| Number of scaffolds > 100K nt | 2180 | 2221 | 2894 | 175 | 2744 |
| Number of scaffolds > 1M nt | 8 | 11 | 32 | 0 | 72 |
| Number of scaffolds > 10M nt | 0 | 0 | 0 | 0 | 0 |
| Mean scaffold size | 53843 | 57956 | 80377 | 16612 | 103266 |
| N50 scaffold length | 100777 | 112528 | 163486 | 27721 | 247286 |
| L50 scaffold count | 2147 | 1879 | 1560 | 7667 | 1018 |
| n90 scaffold length | 23726 | 25264 | 36924 | 7603 | 47949 |
| L90 scaffold count | 8902 | 8218 | 6543 | 25623 | 4533 |
| scaffold %A | 29.65 | 29.63 | 29.62 | 29.66 | 29.62 |
| scaffold %C | 20.36 | 20.37 | 20.36 | 20.35 | 20.36 |
| scaffold %G | 20.35 | 20.36 | 20.37 | 20.34 | 20.36 |
| scaffold %T | 29.64 | 29.64 | 29.65 | 29.65 | 29.63 |
| scaffold %N | 0.00 | 0.00 | 0.00 | 0.00 | 0 |

**Table S2**. Repeated elements in the *Ancistrus triradiatus* genome assembly, obtained through RepeatMasker 4.1.1 (http://www.repeatmasker.org).

|  | Number of elements | Occupied length (bp) | Percentage of the genome sequence |
| --- | --- | --- | --- |
|  |  |  |  |
| **Retroelements:** | 83951 | 29134538 | 2,94 |
| **SINEs:** | 0 | 0 | 0.00 |
| Penelope | 10511 | 2922132 | 0.29 |
| **LINEs:** | 68445 | 22594122 | 2,28 |
| CRE/SLACS | 0 | 0 | 0.00 |
| L2/CR1/Rex | 44340 | 14737387 | 1,49 |
| R1/LOA/Jockey | 4724 | 1121977 | 0.11 |
| R2/R4/NeSL | 269 | 178651 | 0.02 |
| RTE/Bov-B | 6662 | 3312394 | 0.33 |
| L1/CIN4 | 948 | 288010 | 0.03 |
| **LTR elements:** | 15506 | 6540416 | 0.66 |
| BEL/Pao | 34 | 11202 | 0.00 |
| Ty1/Copia | 0 | 0 | 0.00 |
| Gypsy/DIRS1 | 14133 | 5310280 | 0.54 |
| Retroviral | 1250 | 1101873 | 0.11 |
| **DNA transposons:** | 337895 | 67853934 | 6,84 |
| hobo-Activator | 23812 | 5142136 | 0.52 |
| Tc1-IS630-Pogo | 296972 | 59064298 | 5,95 |
| En-Spm | 0 | 0 | 0.00 |
| MuDR-IS905 | 0 | 0 | 0.00 |
| PiggyBac | 459 | 138450 | 0.01 |
| Tourist/Harbinger | 3176 | 636465 | 0.06 |
| Other (Mirage,P-element,Transib) | 373 | 161685 | 0.02 |
| Rolling-circles | 0 | 0 | 0.00 |
| **Unclassified:** | 1346830 | 234942914 | 23.68 |
| **Small RNA:** | 0 | 0 | 0.00 |
| **Satellites:** | 0 | 0 | 0.00 |
| **Simple repeats:** | 8432 | 1263074 | 0.13 |
| **Low complexity:** | 981 | 107549 | 0.01 |
| **Total interspersed repeats** | 331931386 bp |  | 33.45 % |

**Table S3**. Determining the presence or absence of members of the opsin gene family in the genome of *Ancistrus triradiatus*. In total, 23 members of the opsin gene family were examined. The query sequence (first column) is the protein sequence of *Silurus meridionalis* [S.m], and for the genes that are absent in this species, we used the protein sequence of *Danio rerio* [D.r]. Tblastn (with a threshold E-value of 1E-10) was performed against the *Ancistrus triradidatus* genome assembly, and we report the five best scores (E-values) in the second column, and their genomic position (scaffold) on the *A. triradiatus* genome in the third column. Because the opsin gene family has many members with similar sequences, and because some genes are more conserved than others, most blast comparisons gave good hits with several putative opsin genes in *A. triradiatus,* located on different scaffolds*.* To identify the best correspondence, the resulting best scores were crosschecked with the scores of all the other opsins tested, and the overall best pair of [query sequence *versus* scaffold position in *A. triradiatus*], as determined by the best E-value, was considered for the assignation of the gene name. The final gene names are reported in the fourth column of this table. For each query sequence, the best correspondence in the *A. triradiatus* genome assembly is highlighted in bold. To confirm the gene name assignation in the genome of *A. triradiatus*, we looked at the blast results of the assigned sequence and took the assembled tblastn HSPs sequences of *A. triradiatus* (in amino acids) and used blastp to compare it to the NCBI non-redundant protein collection of all actinopterygian species (NCBI, accession date 29.03.2022). The results of this confirmation blastp are given in the two last columns of the table, in which we report the three best annotated hits together with the name of the species and the E-values. We can see that the results of the confirmation blastp converged to the same gene name as given in our initial assignation, highlighted by the green frame in the table.

| **Query** | **evalue (tblastn)** | **Scaffold (*Ancistrus*)** | **Best tblastn correspondance** | **Confirmation blastp** | **evalue (blastp)** |
| --- | --- | --- | --- | --- | --- |
| Rhodopsin 1.1 |  |  |  |  |  |
| [S.m] | **0** | **185** | **rh 1.1** | rhodopsin [*Silurus meridionalis*] | 0 |
|  | 1,33E-110 | 67 | rh 1.2 | rhodopsin [*Tachysurus fulvidraco*] | 0 |
|  | 4,22E-55 | 1189 | exorh | rhodopsin [*Pangasianodon hypophthalamus*] | 0 |
|  | 1,70E-38 | 123 | rh 2 |  |  |
|  | 3,72E-33 | 5068 | opsin 8 |  |  |
| Rhodopsin 1.2 |  |  |  |  |  |
| [S.m] | **9,64E-145** | **67** | **rh 1.2** | rh1.2 (blue-sensitive opsin-like) [*Tachysurus fulvidraco*] | 5,00E-158 |
|  | 1,21E-111 | 185 | rh 1.1 | rh1.2 (blue-sensitive opsin) [*Bagarius yarrelli*] | 1,00E-156 |
|  | 3,00E-42 | 1189 | exorh | rhodopsin like [*Pangasianodon hypophthalamus*] | 2,00E-156 |
|  | 2,18E-31 | 123 | rh 2 |  |  |
|  | 5,43E-22 | 5068 | opsin 8 |  |  |
| Rhodopsin 2 |  |  |  |  |  |
| [S.m] | 3,63E-123 | 185 | rh 1.1 | rh2 (green-sensitive opsin-3) [*Pangasianodon hypophthalmus*] | 4,00E-77 |
|  | 9,22E-92 | 67 | rh 1.2 | rh2 (green-sensitive opsin-3) [*Clarias magur*] | 1,00E-76 |
|  | **6,29E-89** | **123** | **rh 2** | rh2 (green-sensitive opsin-3) [*Colossoma macropomum*] | 1,00E-76 |
|  | 8,86E-41 | 1189 | exorh |  |  |
|  | 3,04E-31 | 561 | lws |  |  |
| Short-wave-sensitive 1 (sws1) | No ortholog found | | |  |  |
| [D.r] | 6,44E-68 | 185 | rh 1.1 |  |  |
|  | 6,75E-56 | 561 | lws |  |  |
|  | 1,40E-47 | 67 | rh 1.2 |  |  |
|  | 1,33E-33 | 123 | rh 2 |  |  |
|  | 9,44E-31 | 154 | parapinopsin |  |  |
| Short-wave-sensitive 2 (sws2) | No ortholog found | | |  |  |
| [D.r] | 4,09E-82 | 185 | rh 1.1 |  |  |
|  | 3,83E-51 | 67 | rh 1.2 |  |  |
|  | 3,10E-45 | 561 | lws |  |  |
|  | 5,47E-26 | 5068 | opsin 8 |  |  |
|  | 4,79E-22 | 123 | rh 2 |  |  |
| Long-wave-sensitive opsin (lws) |  |  |  |  |  |
| [S.m] | **1,67E-82** | **561** | **lws** | long wavelength senstiive opsin [*Anabas testudineus*] | 5,00E-58 |
|  | 1,29E-68 | 185 | rh 1.1 | Long-wave-sensitive opsin (red-sensitive opsin-like) [*Scleropages formosus*] | 4,00E-56 |
|  | 3,99E-48 | 67 | rh 1.2 | Long-wavelength opsin 1 [*Eretmobrycon gonzalezi*] | 7,00E-56 |
|  | 1,95E-33 | 154 | parapinopsin |  |  |
|  | 8,66E-30 | 5068 | opsin 8 |  |  |
| Exorh (rhodopsin-like) |  |  |  |  |  |
| [D.r] | 6,45E-143 | 185 | rh 1.1 | rhodopsin [*Astyanax mexicanus*] | 4E-103 |
|  | 3,46E-106 | 67 | rh 1.2 | rhodopsin-like [*Seriola lalandi dorsalis*] | 7E-103 |
|  | **6,50E-66** | **1189** | **exorh** | rhodopsin-like [*Colossoma macropomum*] | 8E-103 |
|  | 1,02E-63 | 123 | rh 2 |  |  |
|  | 8,40E-33 | 154 | parapinopsin |  |  |
| Vertebrate ancient long opsin a (valopa) |  |  |  |  |  |
| [S.m] | **1,46E-54** | **1790** | **valopa** | opsin-VA-like isoform X1 [*Astyanax mexicanus*] | 3,00E-140 |
|  | 3,46E-52 | 185 | rh 1.1 | vertebrate ancient long opsin a [*Colossoma macropomum*] | 4,00E-140 |
|  | 5,69E-45 | 7475 | valopb | vertebrate ancient long opsin a isoform X1 [*Pygocentrus nattereri*] | 7,00E-139 |
|  | 6,10E-45 | 933 | ? |  |  |
|  | 4,77E-37 | 154 | parapinopsin |  |  |
| Vertebrate ancient long opsin b (valopb) |  |  |  |  |  |
| [S.m] | **3,71E-60** | **7475** | **valopb** | vertebrate ancient long opsin b [*Pangasianodon hypophthalmus*] | 5,00E-68 |
|  | 8,57E-60 | 933 | ? | vertebrate ancient long opsin b [*Ictalurus punctatus*] | 3,00E-67 |
|  | 4,44E-59 | 185 | rh 1.1 | vertebrate ancient opsin-like [*Tachysurus fulvidraco*] | 1,00E-65 |
|  | 1,29E-42 | 154 | parapinopsin |  |  |
|  | 6,84E-41 | 1790 | valopa |  |  |
| Parapinopsin a |  |  |  |  |  |
| [S.m] | **6,67E-105** | **154** | **parapinopsin** | parapinopsin [*Clarias magur*] | 1,00E-177 |
|  | 1,10E-68 | 185 | rh 1.1 | parapinopsin a [*Colossoma macropomum*] | 2,00E-177 |
|  | 1,00E-39 | 67 | rh 1.2 | parapinopsin a [*Pygocentrus nattereri*] | 1,00E-176 |
|  | 2,12E-34 | 5068 | opsin 8 |  |  |
|  | 1,28E-25 | 561 | lws |  |  |
| Parietopsin |  |  |  |  |  |
| [S.m] | **1,45E-152** | **759** | **parietopsin** | parietopsin [*Ictalurus punctatus*] | 2,00E-167 |
|  | 3,84E-33 | 1216 | ? | Tachykinin-like peptides receptor 86C [*Bagarius yarrelli*] | 3,00E-147 |
|  |  |  |  | parietopsin [*Silurus meridionalis*] | 3,00E-144 |
| Opsin 3 |  |  |  |  |  |
| [S.m] | **4,71E-60** | **3543** | **opsin 3** | opsin-3 isoform X2 [Perca flavescens] | 6,00E-57 |
|  | 1,97E-42 | 5068 | opsin 8 | opsin-3 isoform X2 [*Micropterus dolomieu*] | 2,00E-56 |
|  | 7,28E-37 | 185 | rh 1.1 | opsin-3 [*Silurus meridionalis*] | 3,00E-56 |
|  | 1,61E-20 | 67 | rh 1.2 |  |  |
|  | 1,08E-17 | 769 | parietopsin |  |  |
| Teleost multiple tissue opsin a (tmtopsa) |  |  |  |  |  |
| [S.m] | **1,55E-49** | **3568** | **tmtopsa** | teleost multiple tissue opsin a isoform X3 [*Pangasianodon hypophthalmus*] | 3,00E-55 |
|  | 2,71E-42 | 2765 | tmtopsb | teleost multiple tissue opsin a isoform X2 [*Pangasianodon hypophthalmus*] | 1,00E-54 |
|  | 3,64E-38 | 185 | rh 1.1 | teleost multiple tissue opsin a isoform X2 [*Silurus meridionalis*] | 2,00E-54 |
|  | 1,59E-33 | 5068 | opsin 8 |  |  |
|  | 8,37E-23 | 67 | rh 1.2 |  |  |
| Teleost multiple tissue opsin b (tmtopsb) |  |  |  |  |  |
| [S.m] | **8,48E-44** | **2765** | **tmtopsb** | teleost multiple tissue opsin b [*Colossoma macropomum*] | 0 |
|  | 3,43E-39 | 3568 | tmtopsa | teleost multiple tissue opsin b isoform X1 [*Pygocentrus nattereri*] | 0 |
|  | 1,53e,38 | 185 | opsin 8 | pinopsin-like [*Astyanax mexicanus*] | 0 |
|  | 3,15E-37 | 5068 | rh 1.1 |  |  |
|  | 3,96E-27 | 67 | rh 1.2 |  |  |
| Teleost multiple tissue opsin 2a (tmtops2A) | No ortholog found | | |  |  |
| [D.r] | 1,23E-51 | 255 | tmtops2b |  |  |
|  | 2,60E-42 | 5068 | opsin 8 |  |  |
|  | 3,78E-42 | 185 | rh 1.1 |  |  |
|  | 1,50E-31 | 3568 | tmtopsa |  |  |
|  | 1,46E-30 | 2765 | tmtopsb |  |  |
| Teleost multiple tissue opsin 2b (tmtops2B) |  |  |  |  |  |
| [S.m] | **5,59E-51** | **255** | **tmtops2b** | teleost multiple tissue opsin 2b [*Pygocentrus nattereri*] | 1,00E-163 |
|  | 1,45E-36 | 5068 | opsin 8 | vertebrate ancient opsin-like [*Astyanax mexicanus*] | 2,00E-162 |
|  | 1,02E-35 | 185 | rh 1.1 | teleost multiple tissue opsin 2b [*Colossoma macropomum*] | 2,00E-162 |
|  | 5,78E-32 | 2765 | tmtopsb |  |  |
|  | 7,64E-32 | 3568 | tmtopsa |  |  |
| Teleost multiple tissue opsin 3a (tmtops3A) | No ortholog found | | |  |  |
| [D.r] | 1,53E-34 | 185 | rh 1.1 |  |  |
|  | 7,68E-32 | 5068 | opsin 8 |  |  |
|  | 6,95E-29 | 3568 | tmtopsa |  |  |
|  | 9,65E-26 | 2765 | tmtopsb |  |  |
|  | 4,56E-22 | 255 | tmtops2b |  |  |
| Teleost multiple tissue opsin 3b [tmtops3b] | No ortholog found | | |  |  |
| [D.r] | 3,65E-29 | 185 | rh 1.1 |  |  |
|  | 1,64E-26 | 5068 | opsin 8 |  |  |
|  | 2,05E-26 | 3568 | tmtopsa |  |  |
|  | 3,24E-26 | 255 | tmtops2b |  |  |
|  | 3,25E-26 | 2765 | tmtopsb |  |  |
| Opsin 6 |  |  |  |  |  |
| [S.m] | **7,22E-60** | **6** | **opsin 6** | opsin 6, group member a [*Pangasianodon hypophthalmus*] | 0 |
|  | 4,31E-29 | 185 | rh 1.1 | opsin 6, group member a [*Ictalurus punctatus*] | 0 |
|  | 1,25E-26 | 5068 | opsin 8 | opsin-5-like [*Tachysurus fulvidraco*] | 0 |
|  | 3,56E-23 | 897 | ? |  |  |
|  | 1,97E-20 | 917 | opsin 5 |  |  |
| Opsin 7 |  |  |  |  |  |
| [S.m] | **2,59E-54** | **974** | **opsin 7** | opsin 7, group member b [*Pangasianodon hypophthalmus*] | 4,00E-68 |
|  | 3,97E-31 | 5068 | opsin 8 | opsin-5-like protein [*Labeo rohita*] | 2,00E-66 |
|  | 1,36E-24 | 917 | opsin 5 | opsin 7, group member b isoform X2 [*Pygocentrus nattereri*] | 1,00E-65 |
|  | 3,71E-23 | 185 | rh 1.1 |  |  |
|  | 1,68E-20 | 2260 | ? |  |  |
| Opsin 8 |  |  |  |  |  |
| [S.m] |  |  |  |  |  |
|  | **1,71E-44** | **5408** | **opsin 8** | opsin-5-like [*Pangasianodon hypophthalmus*] | 5,00E-87 |
|  | 4,67E-26 | 917 | opsin 5 | opsin-5-like [*Tachysurus fulvidraco*] | 1,00E-84 |
|  | 2,30E-18 | 5068 | opsin 8 | opsin 8, group member b [*Colossoma macropomum*] | 2,00E-80 |
|  | 8,50E-18 | 185 | rh 1.1 |  |  |
|  | 7,77E-11 | 3568 | tmtopsa |  |  |
| Opsin 9 | No ortholog found | | |  |  |
| [D.r] | 9,64E-30 | 5068 | ? |  |  |
|  | 6,34E-26 | 917 | opsin 5 |  |  |
|  | 9,17E-14 | 185 | rh 1.1 |  |  |
|  | 4,46E-12 | 5408 | opsin 7 |  |  |
| Opsin 5 |  |  |  |  |  |
| [D.r] | **3,28E-80** | **917** | **opsin 5** | opsin-5 isoform X1 [*Pygocentrus nattereri*] | 0 |
|  | 8,84E-35 | 5068 | opsin 8 | opsin-5 [*Colossoma macropomum*] | 0 |
|  | 8,76E-27 | 185 | rh 1.1 | CD2-associated -like protein [*Labeo rohita*] | 0 |
|  | 2,84E-19 | 5408 | opsin 7 |  |  |
|  | 3,29E-17 | 974 | opsin 6 |  |  |

**Table S4**. Predicted amino acid sequences of the four genes belonging to the opsin family in *Ancistrus triradiatus* that we analyzed more specifically: *tmtopsa*, *tmtopsb*, *tmtops2b*, and *opsin 5*. For the *tmtops* genes, the protein sequence of *Silurus meridionalis* was used as query sequence against the genome of *Ancistrus triradiatus* using tBlastN, and the HSPs of the best corresponding sequence in *A. triradiatus* (as explained in table S4) were assembled in the correct order to reconstitute the amino acid sequence of *A. triradiatus*, also guided by a well annotated multiple species sequence alignment and using our transcriptome assembly of *A. triradiatus* for verification. The same procedure was used to reconstitute de amino acid sequence of the *opsin 5* gene, except that the protein query sequence was taken from *Danio rerio* (as the *opsin 5* gene is absent from the genome of *Silurus meridionalis).*

>tmtopsa_Ancistrus_triradiatus

EPQLQLSPTGHLVVASCLGLIASLGFVNNLLALVLFGRHKALRSPINLLLINISLSDMLVCALATPFSLAASTRGRWLAGHTGCVWYGFANSLFGIVSLISLAVLSYERYCTMMCPAEADATNYRKVALGVALSWVYSLIWTLPPFFGWSRYGPEGPGTTCSVDWTAKTTNNISYIICLFVFCLVLPFFVIVYSYGKLLHAIRQVSRLNTAVTRKREQRVLFLVVTMVVCYLLCWLPYGIMALVATFGQPGLVSPEASIIPSLLAKTSTVINPVIYIFMNKQFYRCFRSLLTCDAPQRGSSLRSWSKGNKMALTMRRTDNNLTFMAASVEHTSGPPGQDGISNERASKGHAVMDHERPSDQGQASTDAARAPVLSLVAQYN

>tmtopsb_Ancistrus_triradiatus

MIASNLSCAWCANASDSAGAHLQTEGDRRESERDLSSTGHLIVAVCLGFIGTFGFLNNALVLVLFCRYKLLRSPINCLLASISVSDLLVCVLGTPFSFAASTRGRWLIGAAGCVWYGFINSFLGIVSLISLAVLSYERYCTMMGATQADATSYRKVVMGITFSWIYSMIWTLPPLFGWSRYSPEGPGTTCSVNWTSRTANNVSYIVCLFFFCLILPFFVILYSYGKLLQAIKQVTRINTAVTRKREQRVLFLVVTMVVCYLLCWLPYGIMALVATFGQPGLVSPEASIIPSLLAKTSTVINPVIYIFMNKQFYRCFRALLMCSAIHRGSTYKNSSKCTKTPRTLRRANGPNATIMGATPYPATAINPDSDKPKSNSEPEGHYSGTGMAPGTAKPILSLVAYYNG

>tmtops2b_Ancistrus_triradiatus

MTAEELLDVLDGDRDDSPVPTLSSAGFLTLSIFLGFIMTFGFLNNLIVLVLFCRFKKLRTPMNMLLLNISVSDMLVCVCGTTLSFASSLHGRWLLGRRGCMWYGFINSCFGIVSLISLVILSFDRYSTLTVYNKRRPDYLRPLLAVGGSWFYSLFWTLPPLLGWSSYGLEGAGTSCSVSWKERSPQSHAYIICLFIFCLGLPVLVMVYSYGRLLYTVRKIGKIRKTAAWRKENHLLFMIIIAVVSYMLCWTPYSIVALMATFGPSGIITPVAHVVPSLLAKSSTVINPIIYILLNKQFYRCFLMLFCCKAKYTTDSQSIMASKTTVIQLKGIMQRDTEACTAQICTELQ

>opsin_5_Ancistrus_triradiatus

MDNETSHHTGYIPPYLLRGDPFTSKLSKEADIVAAIYIFVIGGVVSAIGNGYVIYMAIKRKTKLKPPELMTLNLAVFDFGISGKPFFMVSSFNHRWPFGWEGCRFYGWTGFFFGCGSLITMTVVSLERYLKICHLRYGTWVKRQHAMQSILFVWLYAAFWAMMPLVGWGSYAPEPFGTSCTLNWWLAQASVSGQSFVMCMLFFCLILPTVIIVFSYAKIIVKVKSSAKEVSYFDSRNNSRNLEMKLTKVAMLICAGFLLAWIPYAVVSVVSAFGKPDTVPIPVSVIPTLLAKSSAMYNPIIYQVIDCKNSCTKSSSFKALSKKKQYNTQR

**Table S5**. Description of the members of the 10 most significantly expanded gene families in *Ancistrus triradiatus*. The software CAFE5 (Mendes et al. 2020) was used to detect the expanded gene families, and for the 10 gene families displaying the top significance scores, we used NCBI blastp to retrieve a gene name or description for every member of the gene families.

| *Sequence* | *Description* | *e-Value* |
| --- | --- | --- |
| **Family 1** | | |
| FUN_010122-T1 | MHC class I alpha chain | 9.52314E-158 |
| FUN_010123-T1 | MHC class I alpha chain | 4.1941E-156 |
| FUN_010127-T1 | BOLA class I histocompatibility antigen, alpha chain BL3-7-like isoform X1 | 1.65358E-160 |
| FUN_010128-T1 | MHC class I alpha chain | 2.04595E-148 |
| FUN_010129-T1 | BOLA class I histocompatibility antigen, alpha chain BL3-7-like isoform X2 | 1.03607E-63 |
| FUN_010130-T1 | BOLA class I histocompatibility antigen, alpha chain BL3-7-like isoform X1 | 6.06302E-147 |
| FUN_010131-T1 | BOLA class I histocompatibility antigen, alpha chain BL3-7-like isoform X1 | 5.33401E-139 |
| FUN_010132-T1 | BOLA class I histocompatibility antigen, alpha chain BL3-7-like isoform X1 | 1.15476E-135 |
| FUN_010134-T1 | BOLA class I histocompatibility antigen, alpha chain BL3-7-like | 1.90116E-66 |
| FUN_010135-T1 | BOLA class I histocompatibility antigen, alpha chain BL3-7-like isoform X1 | 9.54042E-85 |
| FUN_014431-T1 | MHC class I alpha chain | 2.74742E-71 |
| FUN_014432-T1 | H-2 class I histocompatibility antigen, Q9 alpha chain-like | 3.57829E-101 |
| FUN_016693-T1 | BOLA class I histocompatibility antigen, alpha chain BL3-7-like | 8.77327E-84 |
| FUN_016695-T1 | BOLA class I histocompatibility antigen, alpha chain BL3-7-like | 7.54445E-90 |
| FUN_016698-T1 | H-2 class I histocompatibility antigen, Q10 alpha chain-like isoform X2 | 5.01106E-126 |
| FUN_016699-T1 | MHC class I alpha chain | 6.17126E-117 |
| FUN_018337-T1 | BOLA class I histocompatibility antigen, alpha chain BL3-7-like isoform X1 | 1.07526E-114 |
| FUN_018396-T1 | BOLA class I histocompatibility antigen, alpha chain BL3-7-like isoform X1 | 1.1884E-136 |
| FUN_018404-T1 | MHC class I alpha chain | 4.70735E-94 |
| FUN_020423-T1 | MHC class I alpha chain | 5.4189E-120 |
| FUN_020812-T1 | BOLA class I histocompatibility antigen, alpha chain BL3-7-like | 5.39292E-63 |
| FUN_020813-T1 | H-2 class I histocompatibility antigen, Q10 alpha chain-like | 2.57201E-31 |
| FUN_020814-T1 | BOLA class I histocompatibility antigen, alpha chain BL3-7-like | 7.84232E-89 |
| FUN_020815-T1 | MHC class I antigen | 5.61161E-47 |
| FUN_021210-T1 | H-2 class I histocompatibility antigen, Q10 alpha chain-like isoform X2 | 7.8757E-80 |
| FUN_021211-T1 | H-2 class I histocompatibility antigen, Q10 alpha chain-like isoform X2 | 1.54234E-78 |
| FUN_021212-T1 | H-2 class I histocompatibility antigen, Q10 alpha chain-like isoform X2 | 9.27887E-72 |
| FUN_021213-T1 | MHC class I alpha chain | 2.01889E-124 |
| FUN_022340-T1 | BOLA class I histocompatibility antigen, alpha chain BL3-7-like | 4.52343E-93 |
| FUN_022341-T1 | MHC class I antigen | 1.35519E-39 |
| FUN_022342-T1 | BOLA class I histocompatibility antigen, alpha chain BL3-7-like isoform X2 | 2.87293E-28 |
| FUN_022413-T1 | H-2 class I histocompatibility antigen, Q9 alpha chain-like | 1.91439E-20 |
| FUN_022414-T1 | BOLA class I histocompatibility antigen, alpha chain BL3-7-like | 1.87123E-89 |
| FUN_022507-T1 | BOLA class I histocompatibility antigen, alpha chain BL3-7-like isoform X1 | 3.13882E-84 |
| FUN_022508-T1 | BOLA class I histocompatibility antigen, alpha chain BL3-7-like isoform X1 | 1.76197E-107 |
| FUN_022509-T1 | BOLA class I histocompatibility antigen, alpha chain BL3-7-like isoform X1 | 1.7943E-61 |
| FUN_022510-T1 | BOLA class I histocompatibility antigen, alpha chain BL3-7-like isoform X2 | 7.1366E-74 |
| FUN_023173-T1 | MHC class I alpha chain | 9.68263E-155 |
| FUN_023358-T1 | H-2 class I histocompatibility antigen, Q10 alpha chain-like isoform X2 | 6.27295E-109 |
| FUN_023387-T1 | DLA class I histocompatibility antigen, A9/A9 alpha chain-like isoform X1 | 0.0 |
| FUN_023612-T1 | BOLA class I histocompatibility antigen, alpha chain BL3-7-like | 9.05849E-86 |
| FUN_023686-T1 | BOLA class I histocompatibility antigen, alpha chain BL3-7-like isoform X1 | 5.28272E-44 |
| FUN_024161-T1 | BOLA class I histocompatibility antigen, alpha chain BL3-7-like isoform X1 | 5.69662E-99 |
| FUN_024226-T1 | BOLA class I histocompatibility antigen, alpha chain BL3-7-like | 2.81528E-85 |
| FUN_024227-T1 | H-2 class I histocompatibility antigen, Q10 alpha chain-like isoform X2 | 1.16273E-101 |
| FUN_025127-T1 | H-2 class I histocompatibility antigen, Q10 alpha chain-like isoform X2 | 5.11248E-100 |
| FUN_025189-T1 | hereditary hemochromatosis protein homolog | 3.07973E-101 |
| FUN_026135-T1 | BOLA class I histocompatibility antigen, alpha chain BL3-7-like isoform X1 | 2.09911E-90 |
| FUN_026271-T1 | H-2 class I histocompatibility antigen, Q10 alpha chain-like isoform X1 | 2.49449E-111 |
| FUN_026984-T1 | MHC class I alpha chain | 4.5013E-23 |
| **Family 2** | | |
| FUN_011259-T1 | tyrosine-protein phosphatase non-receptor type substrate 1-like | 9.5317E-48 |
| FUN_011260-T1 | hypothetical protein KOW79_016423 | 4.29226E-19 |
| FUN_011261-T1 | tyrosine-protein phosphatase non-receptor type substrate 1-like | 2.19245E-58 |
| FUN_011262-T1 | hypothetical protein KOW79_016429 | 2.33299E-47 |
| FUN_011263-T1 | hypothetical protein KOW79_016432 | 3.20615E-56 |
| FUN_011265-T1 | hypothetical protein KOW79_016438 | 3.7369E-61 |
| FUN_017147-T1 | tyrosine-protein phosphatase non-receptor type substrate 1-like | 4.64073E-65 |
| FUN_017148-T1 | hypothetical protein KOW79_016435 | 7.76373E-66 |
| FUN_017149-T1 | hypothetical protein KOW79_016435 | 6.14701E-48 |
| FUN_017150-T1 | hypothetical protein KOW79_016435 | 3.62235E-69 |
| FUN_017151-T1 | hypothetical protein KOW79_016456 | 2.12645E-39 |
| FUN_017152-T1 | hypothetical protein KOW79_016428 | 1.98678E-59 |
| FUN_017546-T1 | KV401 protein | 1.5813E-58 |
| FUN_019709-T1 | signal-regulatory protein beta-1-like | 7.27727E-53 |
| FUN_019710-T1 | signal-regulatory protein beta-1-like | 4.88495E-56 |
| FUN_023423-T1 | immunoglobulin light chain | 3.51325E-41 |
| FUN_023518-T1 | hypothetical protein KOW79_016454 | 1.9131E-62 |
| FUN_023519-T1 | hypothetical protein KOW79_016456 | 4.45268E-55 |
| FUN_023776-T1 | KV5A2 protein | 7.66474E-48 |
| FUN_025098-T1 | hypothetical protein KOW79_016451 | 8.35026E-55 |
| FUN_025737-T1 | KV5A2 protein | 2.62817E-47 |
| FUN_026996-T1 | tyrosine-protein phosphatase non-receptor type substrate 1-like | 9.56909E-50 |
| FUN_027101-T1 | signal-regulatory protein beta-1-like | 8.69775E-59 |
| **Family 3** | | |
| FUN_010270-T1 | macrophage mannose receptor 1-like | 2.02149E-59 |
| FUN_011961-T1 | macrophage mannose receptor 1-like protein | 4.29746E-36 |
| FUN_017092-T1 | macrophage mannose receptor 1-like | 1.57855E-89 |
| FUN_017094-T1 | macrophage mannose receptor 1-like | 2.13665E-38 |
| FUN_019311-T1 | macrophage mannose receptor 1-like | 1.67734E-29 |
| FUN_019312-T1 | macrophage mannose receptor 1-like | 8.55754E-71 |
| FUN_019315-T1 | macrophage mannose receptor 1-like | 3.7437E-73 |
| FUN_019316-T1 | macrophage mannose receptor 1-like | 8.25509E-48 |
| FUN_023085-T1 | macrophage mannose receptor 1-like | 2.14204E-73 |
| FUN_024274-T1 | macrophage mannose receptor 1-like | 1.72445E-85 |
| FUN_024275-T1 | macrophage mannose receptor 1-like | 4.62873E-47 |
| FUN_025548-T1 | macrophage mannose receptor 1-like | 5.6676E-49 |
| FUN_025556-T1 | macrophage mannose receptor 1-like | 4.89245E-37 |
| FUN_025557-T1 | macrophage mannose receptor 1-like | 2.9651E-31 |
| FUN_026753-T1 | macrophage mannose receptor 1-like | 6.58541E-48 |
| FUN_011960-T1 | macrophage mannose receptor 1-like | 7.07946E-23 |
| FUN_022971-T1 | secretory phospholipase A2 receptor-like | 9.57721E-37 |
| FUN_015172-T1 | macrophage mannose receptor 1-like | 2.38689E-67 |
| FUN_017303-T1 | macrophage mannose receptor 1-like | 5.92108E-47 |
| FUN_019313-T1 | macrophage mannose receptor 1-like | 6.96156E-59 |
| FUN_019314-T1 | macrophage mannose receptor 1-like | 1.28653E-53 |
| FUN_024509-T1 | macrophage mannose receptor 1-like | 1.58963E-33 |
| FUN_025555-T1 | macrophage mannose receptor 1-like | 6.64815E-39 |
| FUN_026236-T1 | macrophage mannose receptor 1-like | 6.31665E-73 |
| FUN_026237-T1 | macrophage mannose receptor 1-like | 3.67792E-64 |
| FUN_012008-T1 | macrophage mannose receptor 1-like | 1.73173E-46 |
| FUN_016972-T1 | macrophage mannose receptor 1-like | 4.74709E-54 |
| **Family 4** | | |
| FUN_007373-T1 | butyrophilin subfamily 1 member A1-like | 4.21094E-125 |
| FUN_007377-T1 | butyrophilin subfamily 1 member A1-like | 1.96272E-63 |
| FUN_007378-T1 | butyrophilin subfamily 1 member A1-like | 6.99394E-64 |
| FUN_007380-T1 | butyrophilin subfamily 1 member A1-like | 1.96202E-58 |
| FUN_007381-T1 | butyrophilin subfamily 1 member A1-like | 1.33335E-54 |
| FUN_007384-T1 | butyrophilin subfamily 1 member A1-like | 6.08943E-59 |
| FUN_013538-T1 | butyrophilin subfamily 1 member A1-like | 2.68942E-57 |
| FUN_013540-T1 | butyrophilin subfamily 1 member A1-like | 2.74301E-52 |
| FUN_016740-T1 | butyrophilin subfamily 1 member A1-like | 1.84498E-55 |
| FUN_017436-T1 | butyrophilin subfamily 1 member A1-like | 3.55697E-129 |
| FUN_019443-T1 | butyrophilin subfamily 1 member A1-like isoform X2 | 1.40649E-27 |
| FUN_022944-T1 | butyrophilin subfamily 1 member A1-like | 1.79848E-29 |
| FUN_026683-T1 | butyrophilin subfamily 1 member A1-like | 3.4484E-55 |
| FUN_026891-T1 | butyrophilin subfamily 1 member A1-like | 3.83694E-50 |
| FUN_027265-T1 | butyrophilin subfamily 1 member A1-like | 1.29853E-63 |
| **Family 5** | | |
| FUN_000001-T1 | urokinase plasminogen activator surface receptor-like | 9.97405E-56 |
| FUN_000002-T1 | urokinase plasminogen activator surface receptor-like | 5.48274E-23 |
| FUN_000003-T1 | urokinase plasminogen activator surface receptor-like | 1.15762E-78 |
| FUN_002446-T1 | urokinase plasminogen activator surface receptor-like | 1.02386E-12 |
| FUN_002447-T1 | urokinase plasminogen activator surface receptor-like | 8.01962E-11 |
| FUN_002448-T1 | ly-6/neurotoxin-like protein 1 | 3.36856E-11 |
| FUN_002450-T1 | urokinase plasminogen activator surface receptor-like | 5.12449E-15 |
| FUN_002451-T1 | urokinase plasminogen activator surface receptor-like | 1.97501E-15 |
| FUN_002452-T1 | urokinase plasminogen activator surface receptor-like | 5.2314E-15 |
| FUN_002453-T1 | urokinase plasminogen activator surface receptor-like | 1.9189E-17 |
| FUN_002454-T1 | urokinase plasminogen activator surface receptor-like | 2.35133E-15 |
| FUN_002455-T1 | urokinase plasminogen activator surface receptor-like | 3.82836E-55 |
| FUN_023607-T1 | urokinase plasminogen activator surface receptor-like | 9.05338E-58 |
| FUN_023608-T1 | phospholipase A2 inhibitor CNF-like | 5.90815E-59 |
| **Family 6** | | |
| FUN_008546-T1 | gamma-crystallin M2-like | 1.43169E-108 |
| FUN_008547-T1 | gamma-crystallin M2-like | 7.37371E-115 |
| FUN_008548-T1 | gamma-crystallin M2-like | 1.36521E-113 |
| FUN_008549-T1 | gamma-crystallin M2-like | 1.32148E-116 |
| FUN_008550-T1 | gamma-crystallin M2-like | 6.62134E-114 |
| FUN_008551-T1 | gamma-crystallin M2-like | 9.45661E-107 |
| FUN_008552-T1 | gamma-crystallin M2-like | 1.55188E-87 |
| FUN_026516-T1 | gamma-crystallin M2-like | 1.58918E-114 |
| FUN_026517-T1 | gamma-crystallin M2-like | 1.48437E-94 |
| FUN_026518-T1 | gamma-crystallin M2-like | 1.75074E-106 |
| FUN_026519-T1 | gamma-crystallin M2-like | 2.47604E-47 |
| **Family 7** | | |
| FUN_014303-T1 | leukocyte elastase inhibitor-like | 6.57248E-20 |
| FUN_014304-T1 | leukocyte elastase inhibitor-like | 3.62126E-174 |
| FUN_014305-T1 | leukocyte elastase inhibitor-like | 4.30993E-175 |
| FUN_014306-T1 | leukocyte elastase inhibitor-like | 0.0 |
| FUN_014307-T1 | leukocyte elastase inhibitor-like | 0.0 |
| FUN_014308-T1 | leukocyte elastase inhibitor-like protein | 0.0 |
| FUN_014309-T1 | leukocyte elastase inhibitor-like | 0.0 |
| FUN_014310-T1 | leukocyte elastase inhibitor-like | 0.0 |
| FUN_017774-T1 | leukocyte elastase inhibitor-like | 9.3525E-78 |
| FUN_017775-T1 | leukocyte elastase inhibitor-like | 1.6938E-52 |
| FUN_017776-T1 | leukocyte elastase inhibitor-like | 7.30519E-74 |
| FUN_017777-T1 | leukocyte elastase inhibitor-like | 0.0 |
| FUN_017778-T1 | leukocyte elastase inhibitor-like | 0.0 |
| FUN_017779-T1 | leukocyte elastase inhibitor-like | 1.92557E-124 |
| FUN_017780-T1 | leukocyte elastase inhibitor-like | 1.60928E-15 |
| FUN_017781-T1 | leukocyte elastase inhibitor | 6.15443E-39 |
| FUN_024778-T1 | leukocyte elastase inhibitor-like | 0.0 |
| FUN_024779-T1 | leukocyte elastase inhibitor-like | 7.70071E-20 |
| FUN_024780-T1 | leukocyte elastase inhibitor-like | 0.0 |
| FUN_024781-T1 | leukocyte elastase inhibitor-like | 2.33501E-123 |
| FUN_025051-T1 | leukocyte elastase inhibitor | 5.99322E-35 |
| FUN_025052-T1 | leukocyte elastase inhibitor-like | 7.67244E-70 |
| FUN_025053-T1 | leukocyte elastase inhibitor-like | 2.81679E-89 |
| FUN_025054-T1 | leukocyte elastase inhibitor-like | 3.05733E-177 |
| FUN_025055-T1 | leukocyte elastase inhibitor-like | 4.63955E-134 |
| FUN_025056-T1 | leukocyte elastase inhibitor-like | 6.51781E-46 |
| FUN_025880-T1 | leukocyte elastase inhibitor-like | 0.0 |
| FUN_025881-T1 | leukocyte elastase inhibitor-like | 2.86189E-22 |
| FUN_026162-T1 | leukocyte elastase inhibitor-like | 0.0 |
| FUN_026295-T1 | leukocyte elastase inhibitor-like | 2.25536E-168 |
| FUN_026858-T1 | leukocyte elastase inhibitor-like | 3.40724E-141 |
| FUN_026931-T1 | leukocyte elastase inhibitor-like | 0.0 |
| FUN_026932-T1 | leukocyte elastase inhibitor-like | 5.23753E-29 |
| FUN_027290-T1 | leukocyte elastase inhibitor-like | 9.5728E-22 |
| **Family 8** | | |
| FUN_001671-T1 | serum amyloid P-component-like | 7.60943E-88 |
| FUN_001672-T1 | serum amyloid P-component-like | 6.17517E-98 |
| FUN_017708-T1 | pentraxin fusion protein-like | 7.75243E-116 |
| FUN_020760-T1 | serum amyloid P-component-like | 1.41371E-72 |
| FUN_023090-T1 | pentraxin fusion protein-like | 1.32869E-132 |
| FUN_024674-T1 | pentraxin fusion protein-like | 2.80783E-112 |
| FUN_026071-T1 | pentraxin fusion protein-like | 9.85897E-111 |
| FUN_026225-T1 | pentraxin fusion protein-like | 1.85638E-145 |
| FUN_026586-T1 | pentraxin fusion protein-like | 7.44866E-109 |
| FUN_027248-T1 | pentraxin fusion protein-like | 6.75514E-90 |
| **Family 9** | | |
| FUN_018141-T1 | C-type lectin domain family 4 member M-like | 4.51855E-50 |
| FUN_019866-T1 | C-type lectin domain family 4 member M-like | 8.63036E-60 |
| FUN_019868-T1 | C-type lectin domain family 4 member M-like | 5.98913E-101 |
| FUN_019869-T1 | C-type lectin domain family 4 member M-like | 6.404E-76 |
| FUN_022433-T1 | GRIP domain-containing protein RUD3-like | 1.71398E-19 |
| FUN_023859-T1 | CD209 antigen-like protein C | 5.42655E-25 |
| FUN_023981-T1 | C-type lectin domain family 4 member M-like isoform X2 | 3.27553E-46 |
| FUN_024178-T1 | C-type lectin domain family 4 member M-like | 1.93816E-81 |
| FUN_025897-T1 | C-type lectin domain family 4 member M-like | 4.51351E-108 |
| FUN_026043-T1 | C-type lectin domain family 4 member M-like isoform X3 | 5.06645E-20 |
| **Family10** | | |
| FUN_006879-T1 | MORC family CW-type zinc finger protein 3-like isoform X2 | 7.30526E-28 |
| FUN_009637-T1 | MORC family CW-type zinc finger protein 3-like | 0.0 |
| FUN_009638-T1 | MORC family CW-type zinc finger protein 3-like | 4.88258E-76 |
| FUN_009640-T1 | MORC family CW-type zinc finger protein 3-like | 1.73437E-41 |
| FUN_019786-T1 | MORC family CW-type zinc finger protein 3-like | 1.02851E-151 |
| FUN_019788-T1 | MORC family CW-type zinc finger protein 3-like | 1.1419E-114 |
| FUN_019789-T1 | MORC family CW-type zinc finger protein 3-like | 6.43423E-100 |
| FUN_019790-T1 | MORC family CW-type zinc finger protein 3-like isoform X2 | 1.6203E-35 |
| FUN_019791-T1 | MORC family CW-type zinc finger protein 3-like isoform X1 | 4.13149E-36 |
| FUN_021155-T1 | MORC family CW-type zinc finger protein 3-like isoform X2 | 6.62424E-127 |
| FUN_021804-T1 | MORC family CW-type zinc finger protein 3-like isoform X1 | 1.37579E-60 |
| FUN_021805-T1 | MORC family CW-type zinc finger protein 3-like | 9.98713E-57 |
| FUN_021806-T1 | MORC family CW-type zinc finger protein 3-like isoform X2 | 1.69676E-126 |
| FUN_023318-T1 | MORC family CW-type zinc finger protein 3 | 0.0 |
| FUN_024034-T1 | MORC family CW-type zinc finger protein 3-like | 3.09609E-41 |
| FUN_024232-T1 | MORC family CW-type zinc finger protein 3-like | 0.0 |
| FUN_024983-T1 | MORC family CW-type zinc finger protein 3-like isoform X2 | 1.09181E-96 |
| FUN_025720-T1 | MORC family CW-type zinc finger protein 3-like | 2.65784E-92 |
| FUN_025823-T1 | MORC family CW-type zinc finger protein 3-like isoform X1 | 6.71563E-36 |
| FUN_025848-T1 | MORC family CW-type zinc finger protein 3-like isoform X1 | 1.48916E-160 |
| FUN_026022-T1 | MORC family CW-type zinc finger protein 3-like | 3.27894E-22 |
| FUN_026343-T1 | MORC family CW-type zinc finger protein 3-like isoform X2 | 2.63134E-85 |
| FUN_026375-T1 | MORC family CW-type zinc finger protein 3-like | 1.13508E-46 |
| FUN_026376-T1 | MORC family CW-type zinc finger protein 3-like isoform X2 | 1.98374E-67 |
| FUN_026549-T1 | MORC family CW-type zinc finger protein 3 | 8.23118E-25 |

**Fig. S1**. Flowchart depicting the steps performed before obtaining the final genome assembly. Wengan (v.02; Di Genova *et al.* 2020) and Haslr (v.0.8; Haghshenas et al. 2020) were used to perform the initial assembly. The WenganD draft assembly was used for the further steps. L_RNA_Scaffolder (Xue et al. 2019) was used to improve the assembly using transcriptomic information, and Kraken2 (v.2.1.1; Lu & Salzberg 2020) to remove potential contamination.


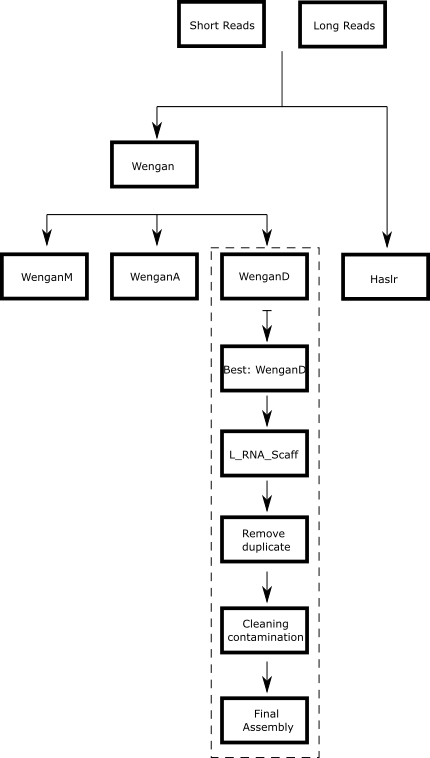


**Fig. S2**. Positive linear regression between the estimated genome size and the predicted number of protein-coding genes in Siluriformes. This corelation analysis is based on the eight catfish genomes reported to date plus the genome of *Ancistrus triradiatus* presented in this paper. The regression was calculted by setting the Y intercept at 0.

**Fig S3**. Maximum-likelihood tree of the protein sequences of the *teleost multiple tissue opsin* (*tmtops*) genes from catfish species and selected relatives. The tree clearly shows the organization into three groups, with intra-group additional gene duplications and gene losses. In the genome of the catfish species (highlighted in grey) *Silurus meridionalis, Ictalurus punctatus, Tachysurus fulvidraco Pangasianodon hypophthalmus* and *Ancistrus triradiatus*, two paralogous copies of the tmtops1 group are present (*tmtopsa* and *tmtopsb*), one copy of the tmtops2 group is present (*tmtops2b*) and no copies of the tmtops3 group are present. This result clarifies the naming of the *tmtops* genes present in the catfish genomes, which was confusing in Zheng *et al*. (2021). The three groups of *tmtops* shown in the phylogenetic tree are in accordance with Sakai *et al* (2015). The tree was obtained based on the analysis of amino acid sequences, with the JTT+G substitution matrix (LnL= -12922.64), and using MEGA XI software (Tamura et al. 2021). All *tmtops* gene copies available on NCBI were used for the selected species, in addition to the genes identified in the genome of *Ancistrus triradiatus.*The accession numbers are as follows: for *Danio rerio* (*tmtopsa*: NP_001112371.1; *tmtopsb*: NP_001299608.1; *tmtops2a*: NP_001268434.1; *tmtops2b*: NP_001269302.1; *tmtops3a*: NP_001269303.1; *tmtops3b*: AGK25000.1), for *Silurus meridionalis* (*rhodopsin*: XP_046726778.1; *tmtopsa*: XP_017350454.1; *tmtopsb*: XP_046733396.1; *tmtops2b*: XP_046698609.1), for *Ictalurus punctatus* (*tmtopsa*: XP_017350454.1; *tmtopsb*: XP_017308205.1; *tmtops2b*: XP_017326070.1), for *Tachysurus fulvidraco* (*tmtopsa*: XP_027014049.1; *tmtopsb*: XP_027020402.1; *tmtops2b*: XP_027029546.1), for *Pangasianodon hypophthalmus* (*tmtopsa*: XP_026801960.1; *tmtopsb*: XP_026793806.1; *tmtops2b*: XP_026768267.2), for *Solea senegalensis* (*tmtopsb*: XP_043901541.1*; tmtops2b*: XP_043873226.1; *tmtops3a*: XP_043895712.1), for *Cyprinodon tularosa* (*tmtopsb*: XP_038123363.1*; tmtops2b*: XP_038145555.1; *tmtops3a*: XP_038145404.1), for *Periophthalmus magnuspinnatus* (*tmtops2b*: XP_033842730.1*; tmtops3a* : XP_033829647.1), and finally, the three *tmtops* protein sequences of *Ancistrus triradiatus* presented in this study (Table S5).


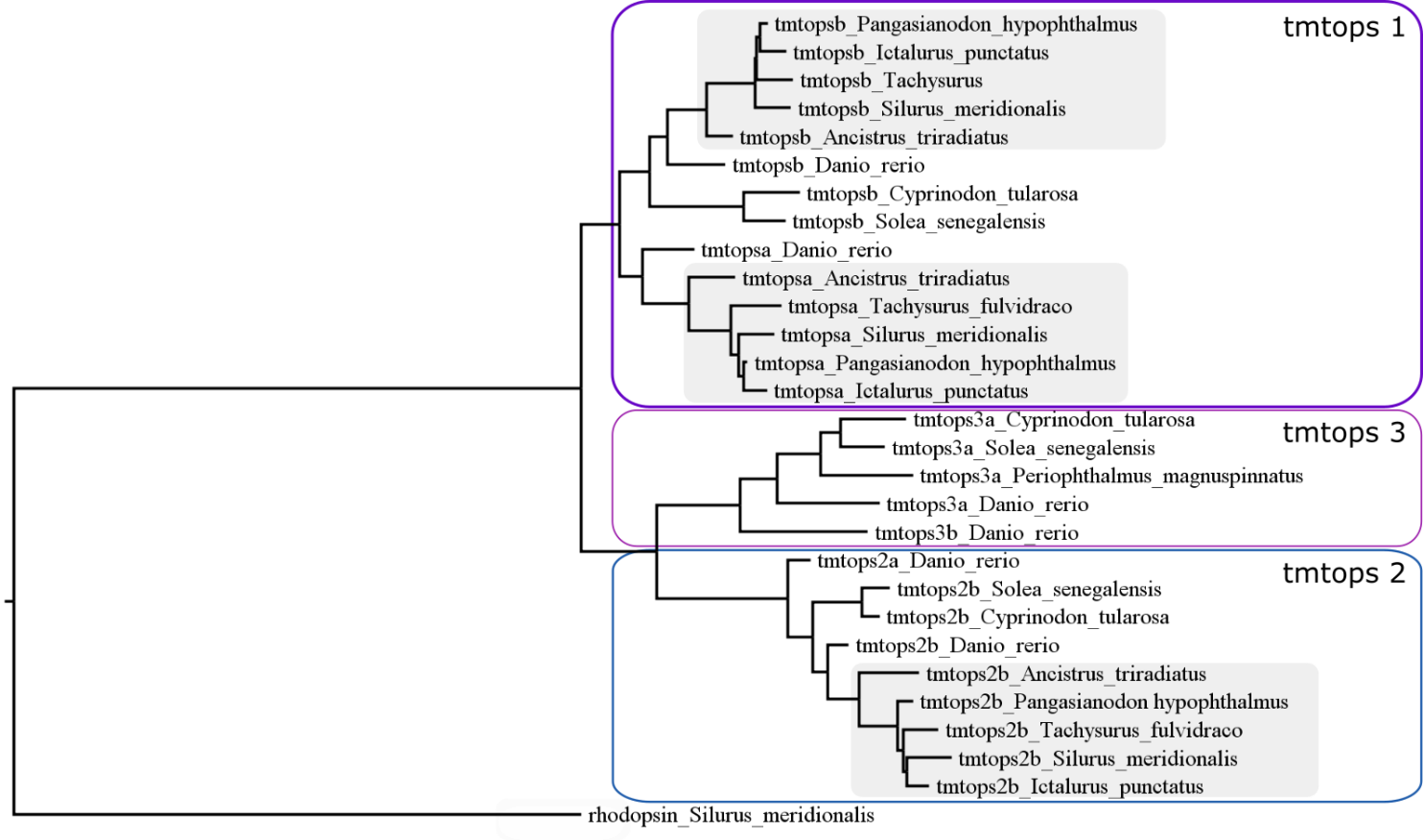


**References**

Di Genova A, Buena-Atienza E, Ossowski S, Sagot M-F (2020) Efficient hybrid de novo assembly of human genomes with WENGAN. Nat Biotechnol. https://doi.org/10.1038/s41587-020-00747-w

Haghshenas E, Asghari H, Stoye J, et al (2020) HASLR: Fast Hybrid Assembly of Long Reads. iScience 23:101389. https://doi.org/10.1016/j.isci.2020.101389

Lu J, Salzberg SL (2020) Ultrafast and accurate 16S rRNA microbial community analysis using Kraken 2. Microbiome 8:1–11. https://doi.org/10.1186/s40168-020-00900-2

Mendes FK, Vanderpool D, Fulton B, Hahn MW (2020) CAFE 5 models variation in evolutionary rates among gene families. Bioinformatics 36:5516–5518. https://doi.org/10.1093/bioinformatics/btaa1022

Tamura K, Stecher G, Kumar S (2021) MEGA11: Molecular Evolutionary Genetics Analysis Version 11. Mol Biol Evol 38:3022–3027. https://doi.org/10.1093/molbev/msab120

Xue W, Li J-T, Zhu Y-P, et al (2019) L_RNA_scaffolder: scaffolding genomes with transcripts. BMC Genomics 20:1–14. https://doi.org/10.1186/s12864-019-5856-1
